# Adaptive metal ion transport and metalloregulation-driven differentiation in pluripotent synthetic cells

**DOI:** 10.1038/s41557-024-01682-y

**Published:** 2024-12-23

**Authors:** Sayuri L. Higashi, Yanjun Zheng, Taniya Chakraborty, Azadeh Alavizargar, Andreas Heuer, Seraphine V. Wegner

**Affiliations:** 1 Institute of Physiological Chemistry and Pathobiochemistry, University of Münster, Münster, Germany; 2 Institute for Advanced Study, Gifu University, Gifu, Japan; 3 Center for One Medicine Innovative Translational Research, Gifu University, Gifu, Japan; 4 United Graduate School of Drug Discovery and Medical Information Sciences, Gifu University, Gifu, Japan; 5 Institute for Physical Chemistry, University of Münster, Münster, Germany

**Keywords:** Synthetic biology, Metalloproteins

## Abstract

Pluripotent cells can yield different cell types determined by the specific sequence of differentiation signals that they encounter as the cell activates or deactivates functions and retains memory of previous inputs. Here, we achieved pluripotency in synthetic cells by incorporating three dormant apo-metalloenzymes such that they could differentiate towards distinct fates, depending on the sequence of specific metal ion transport with ionophores. In the first differentiation step, we selectively transported one of three extracellular metal ion cofactors into pluripotent giant unilamellar vesicles (GUVs), which resulted in elevation of intracellular pH, hydrogen peroxide production or GUV lysis. Previously added ionophores suppress transport with subsequent ionophores owing to interactions among them in the membrane, as corroborated by atomistic simulations. Consequently, the addition of a second ionophore elicits a dampened response in the multipotent GUV and a third ionophore results in no further response, reminiscent of a terminally differentiated GUV. The pluripotent GUV can differentiate into five final fates, depending on the sequence in which the three ionophores are added.

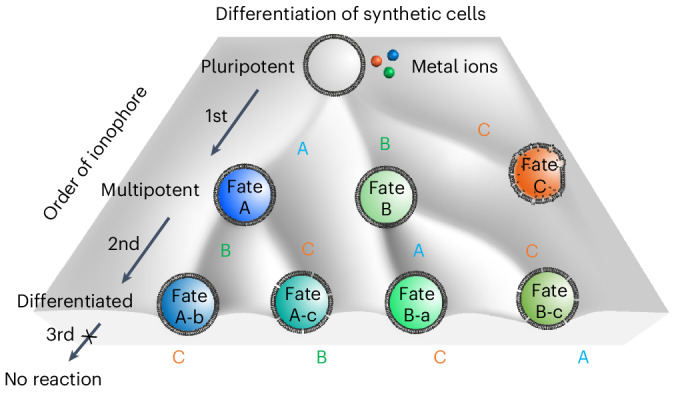

## Abstract (web summary)

The sequence of specific differentiation signals determines the fate of a pluripotent cell. Here pluripotency was introduced into synthetic cells by loading them with three dormant apo-metalloenzymes, which were activated through selective metal ion transport by one of three ionophores. Depending on the sequence of metal ion intake, the synthetic cells differentiated towards five distinct fates.

## Main

Cells possess an extraordinary capacity to respond and adapt to changes in their surroundings by activating specific pathways and retaining a memory of prior inputs^1^. Cell differentiation stands out as an exceptional illustration of this adaptability. Initially identical pluripotent cells progressively acquire new functionalities while concurrently suppressing other dormant ones. Throughout the course of differentiation, pluripotent cells progressively commit to a specific fate, influenced by the temporal sequence of various signals in the extracellular environment and the memory encoded in gene networks and posttranslational processes^2^. As a result, they gradually lose their plasticity, transitioning first into multipotent and ultimately into terminally differentiated cells that no longer respond to previous differentiation cues.

Synthetic cells, assembled from molecular components, are already able to mimic various functions associated with living cells^3,4^, encompassing but not limited to growth^5^, division^5–9^, reproduction^10–12^, differentiation^13^, metabolism^14,15^, communication^16^ and social interactions within communities of synthetic and living cells^17–21^. However, replicating the adaptive multistage differentiation observed in pluripotent cells remains elusive. Towards this goal, lipid-based synthetic cells with cell-free protein expression have been differentiated in multiple steps through the activation of embedded gene circuits with vesicle fusion^13^ and along diffusion gradients formed in emulsion-based compartments^22^. Moreover, non-lipid-based protocells can replicate mechanisms of multicellular differentiation by altering their morphology, membrane permeability and enzymatic activity when exposed to unidirectional or counter-directional chemical gradients^23^. In the context of differentiation in synthetic cells, most examples hinge on cell-free protein synthesis and the differential expression of proteins depending on vesicle fusion^13^ or diffusion gradients of membrane-permeable small molecules^22^. Moreover, the morphological differentiation of coacervate-based synthetic protocells has been achieved along artificial morphogen gradients without relying on protein production^23^. Beyond differentiation in synthetic cells, examples of adaptive behaviour are found only in the context of morphological alterations^24,25^, positioning in the environment^26^ and cell-to-cell communication^27,28^. To achieve a pluripotent synthetic cell that can differentiate towards various fates would require one to orthogonally activate different functions within a single synthetic cell and have these co-regulate each other with a gradual loss of plasticity.

During cellular differentiation, signalling events across the membrane assume central importance as cells render themselves insensitive to signals along the way by downregulating the expression of cognate receptors^29^. Crucially, signal transduction across the membrane necessitates high specificity and preferential amplification^30^. Therefore, adaptive membrane transport, wherein transport across the membrane changes with the history of inputs, provides a unique opportunity for mimicking the differentiation of cells. In this context, reported lipid-based synthetic cells have relied on artificial gene circuits through the use of programmable DNA sequences, along with semipermeable substrates and/or non-specific transporters such as α-haemolysin, to trigger specific cellular functions^13,24,31^. Yet, replicating specific and adaptive signalling has not been possible due to the lack of dedicated transmembrane receptors and transporters that could specifically transport molecules and/or differentially activate internal pathways.

**Fig. 1:**
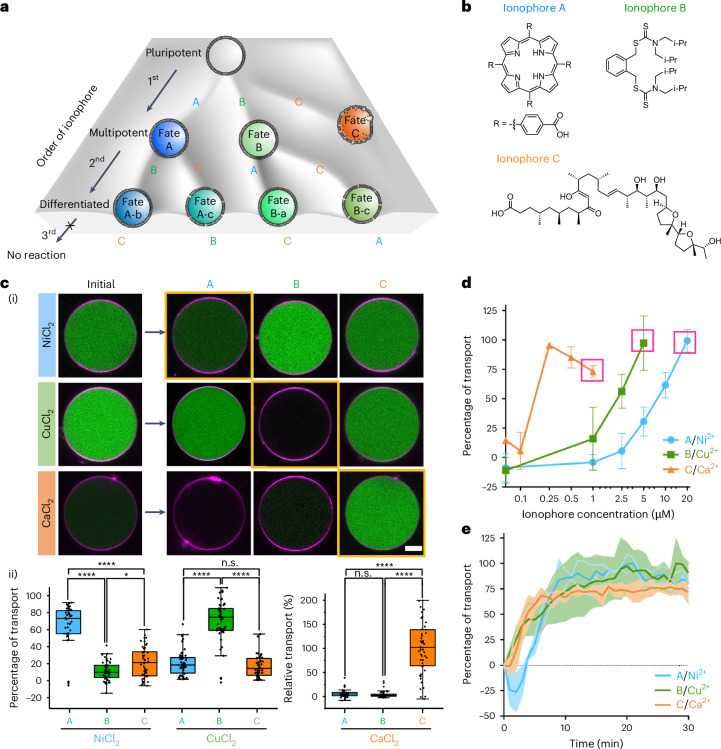
Design of a pluripotent synthetic cell with selective metal ion transport. **a**, A synthetic pluripotent cell differentiates sequentially towards different fates, depending on the sequence in which three different ionophores (A, B and C) are added. Each ionophore activates a certain function through the selective transport of a metal ion in GUVs and affects the response to subsequent ionophores. The commitment to one fate leads to a diminished response to subsequent ionophores. **b**, Chemical structures of each ionophore. **c**, (i) Representative CLSM images of GUVs (membrane shown in red) loaded with [Ca^2+^-Rhod2] (top two rows) or Rhod2 (bottom row) (shown in green) before and 15 min after addition of ionophores A (20 µM), B (5 µM) or C (1 µM) in the presence of external NiCl_2_ (1 µM), CuCl_2_ (1 µM) or CaCl_2_ (100 µM). Scale bar, 10 µm. GUVs in which the fluorescence signal changed, indicative of ion transport, are highlighted with a yellow box. (ii) The relative transport of each metal ion with different ionophores in **b**. *n*_GUV_ = 50. Each GUV is shown as a point in the box plot. Within each box, the horizontal black lines denote median values; the boxes extend from the 25th to the 75th percentile of each group’s distribution of values; the lower and upper boundaries of whiskers indicate the minima and maxima, respectively. Statistical significance is determined by two-tailed non-paired Student’s *t*-tests (****P < 0.0001, ***P < 0.001, **P < 0.01, *P < 0.05; n.s., non-significant). The data were pooled from three independent replicates. **d**,**e**, Ni^2+^, Cu^2+^ and Ca^2+^ transport at different ionophore concentrations (**d**) and kinetics (**e**) with ionophore A (20 µM), B (5 µM) or C (1 µM), respectively, as measured by plate reader. Ionophores were subsequently used at concentrations marked with a pink box. The error bars (with fill area) indicate the s.d. of the average intensities for *n* = 3. Source data

In this work, we demonstrate the selective and adaptive activation of different enzymatic reactions within pluripotent giant unilamellar vesicle (GUV)-based synthetic cells. In particular, we rely on three different metal ions—Ni^2+^, Cu^2+^ and Ca^2+^—as external signals, which are selectively transported into the GUVs with specific ionophores. Subsequently, these metal ions differentially activate apo-metalloenzymes by using these metal ion cofactors. Depending on the specific enzymatic reaction activated, the synthetic cell displays different behaviours, such as increased intracellular pH, hydrogen peroxide (H_2_O_2_) production or cellular lysis. The ionophores serve as decision factors of the cell’s fate, as the first activated enzyme sets the synthetic cell’s fate and suppresses the subsequent activation of the other pathways. The first differentiation step induced by a specific metal ion transport is thereby deterministic, endowing the synthetic cell with specialized capabilities while concurrently losing dormant potential (Fig. 1a).

## Results and discussion

### Selective transport of metal ions into synthetic cells

The specific transport of non-membrane-permeable metal ions into lipid-based vesicles remains a challenge. Although membrane pores (for example, α-haemolysin) let in all molecules below a certain size, they also lead to the loss of encapsulated small molecules. In this study, we proposed the use of ionophores with high specificity for individual metal ions to enable differential transport into GUVs. The ionophores facilitated diffusion along concentration gradients while maintaining separation of intracellular and extracellular content^32,33^. Specifically, we investigated the transport of Ni^2+^ with ionophore A (*meso*-tetraphenylporphine-4,4′,4″,4″′-tetracarboxylic acid)^34^, of Cu^2+^ with ionophore B (*o*-xylylen-bis-(*N,N*-diisobutyldithiocarbamat))^35^ and of Ca^2+^ with ionophore C (ionomycin)^36^ (Fig. 1b).

To determine the specificity of these ionophores in metal ion transport across the membrane, we loaded GUVs (1-palmitoyl-2-oleoyl-glycero-3-phosphocholine (POPC)) with 0.1 mol% DiD (1,1′-dioctadecyl-3,3,3′,3′- tetramethylindodicarbocyanine, 4-chlorobenzenesulfonate salt) membrane dye (shown in red) and the metal ion-sensitive fluorescent dye Rhod2 or its Ca^2+^ complex (shown in green) (both Rhod2 and the complex are membrane impermeable). We monitored the transport of externally added Ni^2+^, Cu^2+^ and Ca^2+^ ions with ionophores A, B or C into the GUVs by using confocal fluorescence microscopy (Fig. 1c). Rhod2 itself is non-fluorescent but becomes fluorescent upon complexing with Ca^2+^ ([Ca^2+^-Rhod2]), which is quenched in the presence of heavy metal cations such as Ni^2+^ or Cu^2+^ ions^37^. The fluorescence of [Ca^2+^-Rhod2] in the GUVs decreased upon addition of ionophore A, but not B or C, in the presence of outer Ni^2+^ ions (Fig. 1c and Supplementary Fig. 1a). Similarly, the addition of ionophore B quenched the [Ca^2+^-Rhod2] fluorescence in the presence of external Cu^2+^ ions, whereas ionophores A and C did not show any effect (Fig. 1c and Supplementary Fig. 1b). The internal fluorescence of Rhod2-loaded GUVs increased in the presence of external Ca^2+^ ions upon addition of ionophore C, whereas no change was observed with ionophores A or B (Fig. 1c and Supplementary Fig. 1c). Ion transport into the GUVs depended on ionophore concentration as measured in bulk populations of GUVs with a plate reader (Fig. 1d and Supplementary Fig. 2a–c). The optimal concentrations for ionophores A, B and C were 20 µM, 5 µM and 1 µM, respectively, and these concentrations were used throughout the study unless otherwise specified. The transport of each ion with its corresponding ionophore was rapid, with changes in Rhod2 fluorescence observed within 5–10 min of ionophore addition in the plate reader measurements (Fig. 1e) and at the level of single GUVs under the microscope (Supplementary Fig. 3a–c). Overall, these findings demonstrate the high selectivity of ionophore A for Ni^2+^, ionophore B for Cu^2+^ and ionophore C for Ca^2+^ for transport into GUVs, other metal ions being excluded.

### Activation of metalloenzymes in synthetic cells

**Fig. 2:**
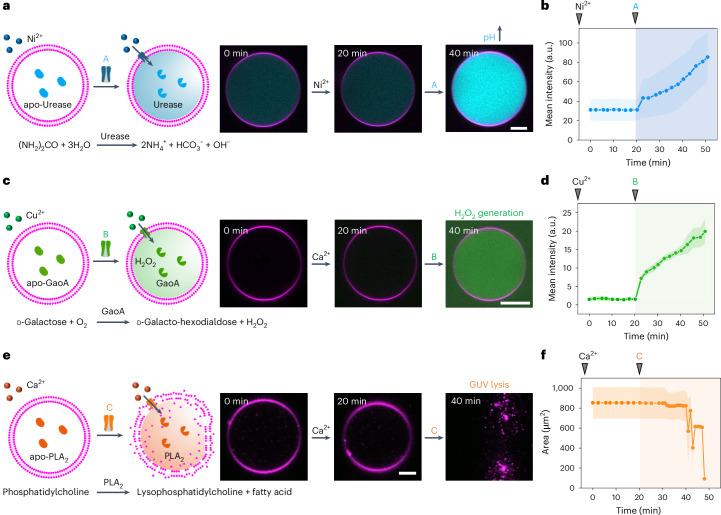
Activation of apo-metalloenzyme through metal ion transport in synthetic cells. **a**, Schematic and CLSM images of the same apo-urease-loaded GUV (membrane shown in red) in the presence of Ni^2+^, increasing its intracellular pH (urease sensor shown in cyan) upon addition of ionophore A owing to Ni^2+^ transport into the GUV and activation of urease. **b**, The fluorescence increase inside GUVs over time in **a**. The error bars with fill area indicate the s.d. of the average intensities for *n*_GUV_ = 10. **c**, Schematic and CLSM images of the same apo-GaoA-loaded GUV in the presence of Cu^2+^, producing H_2_O_2_ (GaoA sensor shown in green) upon addition of ionophore B due to Cu^2+^ transport into the GUV and activation of GaoA. **d**, The fluorescence increase inside the GUV over time in **c**. The error bars with fill area indicate the s.d. of the average intensities for *n*_GUV_ = 10. **e**, Schematic and CLSM images of apo-PLA_2_-loaded GUV, lysing upon addition of ionophore C owing to Ca^2+^ transport and activation of PLA_2_. **f**, The GUV area over time. The error bars with fill area indicate the s.d. of the average intensities for *n*_GUV_ = 3. Scale bars, 10 µm. Individual GUVs were tracked over time in all experiments. Source data

Next, we aimed to translate the selective transport of each metal ion into the activation of distinct enzymatic activities in the GUV. To achieve this, we proposed encapsulating dormant apo-metalloenzymes that become catalytically active upon binding to their cognate metal ion cofactors. In the framework of this study, we chose three metalloenzymes as demonstrators: urease as a Ni^2+^ enzyme that decomposes urea into ammonia and carbon dioxide, resulting in an increased pH^38^; galactose oxidase (GaoA) as a Cu^2+^ enzyme that oxidizes d-galactose to d-galacto-hexodialdose and produces H_2_O_2_ (ref. ^39^); and phospholipase A2 (PLA_2_) as a Ca^2+^ enzyme that cleaves fatty acid chains of phospholipids, leading to the lysis of GUVs^40^. We chose these three enzymes on the basis of multiple requirements. First, our focus was on identifying three metalloenzymes with single metal cofactors that matched the specificity of the ionophores, ruling out metalloenzymes with other metal ion cofactors. Second, we looked for metalloenzymes that had distinct enzymatic activity that could be monitored in real time with available fluorescent sensors. Finally, we verified that the enzymes were stable in their apo-form and not denatured upon metal ion removal. To this end, for each of the three enzymes, we first produced the apo-forms and confirmed the activation of apo-GaoA and apo-urease in solution after addition of their metallocofactor (Supplementary Fig. 4a,b). In addition, we showed that low concentrations of ethylenediaminetetraacetic acid (EDTA, 30 µM), later included in the GUV preparation to avoid aberrant enzyme activation, do not alter enzyme activation upon addition of the metal ions (Supplementary Fig. 4c,d). Next, we evaluated the activation of each apo-metalloenzyme inside the GUV with selective metal ion transport. In GUVs loaded with apo-urease, the fluorescence of the co-encapsulated fluorescent pH indicator, 8-hydroxy-pyrene-1,3,6-trisulfonic acid trisodium salt (HTPS; urease sensor, shown in cyan), was initially low in the presence of the membrane-permeable substrate urea and external Ni^2+^ ions, as observed over 20 min (Fig. 2a). After we added ionophore A, the fluorescence of the urease sensor increased as Ni^2+^ ions entered the GUVs and activated the urease, producing ammonia and increasing the internal pH. The pH in the GUVs rose from 7.4 to above 9 within 20 min, as observed by tracking the same GUV over time (Fig. 2b and Supplementary Fig. 5) and by analysing a population of GUVs after 20 min (Supplementary Fig. 6a). Notably, we observed that few GUVs deformed and even formed interconnected vesicles as a result of elevated internal pH and osmotic pressure, as also reported in some previous studies^6,38,41^ (Supplementary Fig. 7 and Supplementary Movie 1).

To demonstrate the activation of apo-GaoA inside GUVs via Cu^2+^ transport, we loaded apo-GaoA, galactose (membrane impermeable) and horseradish peroxidase (HRP) into GUVs while Cu^2+^ and Amplex red were present in the external solution (Fig. 2c). After 20 min of incubation, the GUVs exhibited no notable increase in their internal fluorescence from the GaoA sensor (shown in green; H_2_O_2_ oxidizes non-fluorescent Amplex red (membrane permeable) to fluorescent resorufin (membrane impermeable) catalysed by HRP). Yet, upon initiation of the Cu^2+^ transport with ionophore B, the fluorescence signal from the GaoA sensor in the GUV increased over 20 min (Fig. 2d). Complementarily, population-level analysis (Supplementary Fig. 6b) and the detection of H_2_O_2_ with a chemiluminescence assay (Supplementary Fig. 8) provided further evidence of increased GaoA activity following the addition of ionophore B.

To demonstrate the activation of apo-PLA_2_, we encapsulated the apo-enzyme into GUVs, which remained stable in the presence of external Ca^2+^ after 20 min (Fig. 2e). On addition of ionophore C, the GUV membranes started to break after approximately 10 min (Fig. 2f and Supplementary Movie 2), eventually leading to the lysis of the GUV into small micelles. In the analysis of multiple GUVs, a visible reduction in the area of GUVs was observed (Supplementary Fig. 6c). In all these experiments, we first added the metal ion and second the ionophore after 20 min equilibration to activate the apo-enzymes encapsulated in the GUVs, but this worked equally well when the ionophore was added first and the metal ion second after 20 min (Supplementary Fig. 9). Moreover, for all three enzymes, control experiments showed that there was no unspecific enzyme activation without added ionophores (Supplementary Fig. 11). These results exemplify the activation of different apo-metalloenzymes with distinct metal ion cofactors within GUVs through selective ionophore-mediated transport, resulting in diverse outcomes, such as pH increase, H_2_O_2_ generation and vesicle lysis.

### Differential enzyme activation in a synthetic cell

**Fig. 3:**
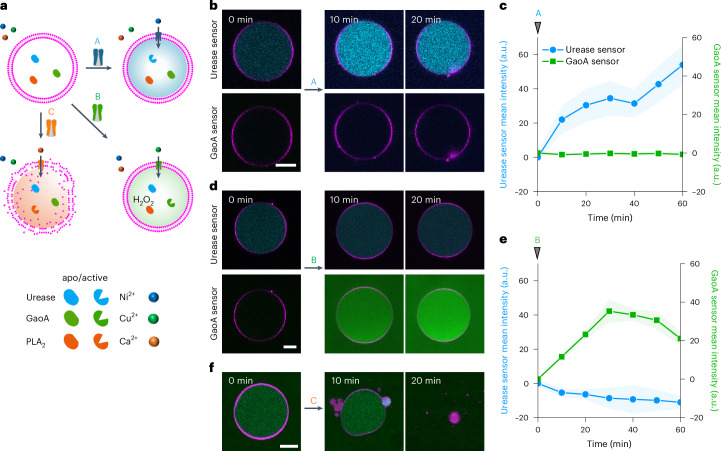
Selective metalloenzyme activation with specific metal ion transport. **a**, Schematic of a pluripotent synthetic cell loaded with three apo-metalloenzymes (apo-urease, apo-GaoA and apo-PLA_2_) differentiated with different ionophores in the presence of three external metal ions (Ni^2+^, Cu^2+^ and Ca^2+^). **b**,**d**,**f**, CLSM images of pluripotent GUV (membrane shown in red) loaded with urease sensor (shown in cyan) and GaoA sensor (shown in green) after adding ionophore A, resulting in a pH increase (**b**); ionophore B, resulting in H_2_O_2_ production (**d**); or ionophore C, resulting in GUV lysis (**f**). Scale bars, 10 µm. **c**,**e**, Fluorescence change for the urease sensor and GaoA sensor inside GUV in **b** (**c**) and in **d** (**e**). The error bars with fill area indicate the s.d. of the average intensities for *n*_GUV_ = 10. Individual GUVs were tracked over time in all experiments. Source data

We next investigated whether the selective activation of a single metalloenzyme is possible in a pluripotent synthetic cell with multiple dormant enzymes. For this purpose, we loaded GUVs with all three apo-enzymes and assessed whether it is possible to activate one specific enzyme with all three metal ions present in the environment by using the corresponding ionophore (Fig. 3a). These GUVs (shown in red) also contained the necessary components for the urease sensor (shown in cyan) and GaoA sensor (shown in green), as well as all the encapsulated and externally added substrates. On addition of the Ni^2+^-selective ionophore A, we observed an increase in the fluorescence signal from the urease sensor, leading to fate A, characterized by an elevated internal pH in the pluripotent synthetic cell (Fig. 3b,c). At the same time, there was no increase in fluorescence from the GaoA sensor, and the GUVs remained stable, indicating the selective activation of urease and not the other enzymes. In contrast, when the Cu^2+^-selective ionophore B was added to the pluripotent GUVs, only the fluorescence signal from the GaoA sensor and not the urease sensor increased, leading to fate B, characterized by the production of H_2_O_2_ (Fig. 3d,e). It is worth noting that the gradual decrease in the GaoA sensor signal inside the GUV and increased background at later stages might be due to the photobleaching of resorufin inside the GUV and the residual activity of enzymes outside the vesicles as well as the non-catalysed photooxidation of Amplex red to resorufin. Finally, upon addition of the Ca^2+^-selective ionophore C to the pluripotent GUVs, the GUVs ruptured over time, leading to fate C, characterized by GUV lysis as a result of the specific activation of PLA_2_ (Fig. 3f and Supplementary Movie 3). Moreover, when we tracked the fluorescence of the urease and GaoA sensors after adding ionophore C, we observed no increase in fluorescence in either channel (Supplementary Fig. 10), presumably because the loaded galactose and the urease sensor leaked from the GUVs. Notably, incubating the pluripotent GUVs solely with the three metal ions failed to induce enzyme activation (Supplementary Fig. 11). Furthermore, we confirmed that the transport of metal ions into GUVs by the specific ionophore remained unaffected in the presence of the other spectator ions (Supplementary Fig. 12). Overall, we successfully demonstrated the ability to control the activation of a specific metalloenzyme within pluripotent GUVs, depending on the metal ion transporter.

### Ion transport capacity depends on ionophore sequence

In pluripotent cells, the operation of different pathways is not independent; instead, there is extensive cross-regulation in which the activation of one pathway can suppress or activate others. Consequently, the temporal sequence of differentiation signals is crucial for determining the final cell fate. In this respect, we investigated whether it is possible to sequentially transport different metal ions independent from each other into a pluripotent GUV, or if there are feedback mechanisms that alter the transport of later metal ions.

**Fig. 4:**
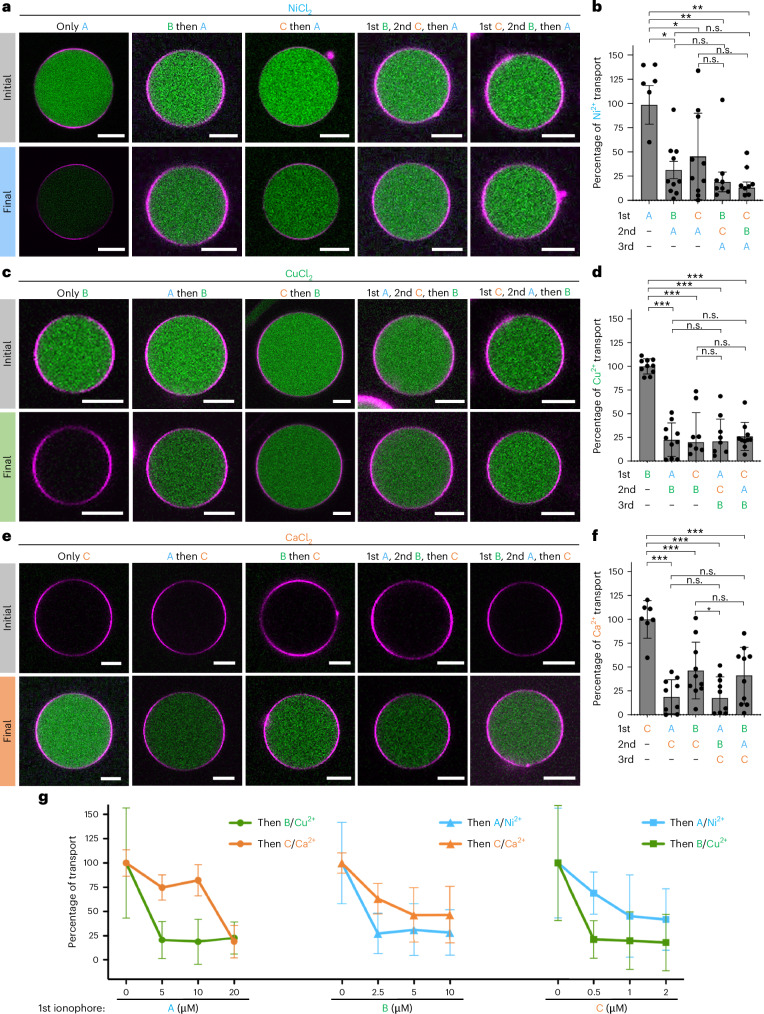
Negative influence of prior ionophores on transport with subsequent ionophores. **a**,**c**,**e**, Representative CLSM images of a GUV (membrane shown in red) loaded with [Ca^2+^-Rhod2] or Rhod2 (shown in green) before and after sequential addition of up to three ionophores (20 µM A, 5 µM B and 1 µM C) in 20 min intervals in the presence of external NiCl_2_ (**a**), CuCl_2_ (**c**) or CaCl_2_ (**e**). Scale bars,10 µm. **b**,**d**,**f**, The relative ion transport of Ni^2+^ in **a** (**b**), Cu^2+^ in **c** (**d**) and Ca^2+^ in **e** (**f**). The error bars indicate the s.d. of the average intensities for *n*_GUV_ = 10. Statistical significance is determined by two-tailed non-paired Student’s *t*-tests (***P < 0.001, **P < 0.01, *P < 0.05; n.s., non-significant). **g**, The relative ion transport of each metal ion in the presence of different competitor ionophores added 20 min before the cognate ionophore (20 µM A, 5 µM B and 1 µM C). The error bars indicate the s.d. of the average intensities for *n*_GUV_ = 10. Randomly picked GUVs were analysed in each sample. Source data

To achieve this, we initially examined the effects of sequentially adding ionophores on the transport of their specific metal ions. As previously demonstrated, ionophore A transports external Ni^2+^ ions into GUVs based on the quenching of [Ca^2+^-Rhod2] fluorescence (shown in green), which was defined as 100% (Fig. 4a). Yet, when GUVs were first exposed to ionophores B or C for 20 min and then to ionophore A, the relative ion transport for Ni^2+^ ions decreased to 31% and 45%, respectively (Fig. 4b and Supplementary Fig. 13a). The impact of spectator ionophores was even more pronounced when ionophore A was added as the third step after two prior 20 min incubation steps with ionophores B and C. In this case, there was almost no Ni^2+^ transport (below 20%), and the diminished Ni^2+^ transport with ionophore A was independent of the type and order in which the spectator ionophores were added beforehand (B and then C, or C and then B). Likewise, the presence of other ionophores influenced the transport of external Cu^2+^ ions with ionophore B. The prior addition of one (A or C) or two ionophores (A and then C, or C and then A) decreased the relative transport below 25% in all cases, compared with when ionophore B was added alone (Fig. 4c,d and Supplementary Fig. 13b). Similarly, the addition of ionophore A and B negatively impacted the transport of Ca^2+^ into GUVs with ionophore C, visualized as an increase in [Ca^2+^-Rhod2] fluorescence (Fig. 4e). The earlier addition of one (A or B) or two ionophores (A and then B, or B and then A) decreased the relative transport below 50% in all cases, compared with when ionophore C was added first (Fig. 4f and Supplementary Fig. 13c). Thus, the order in which the ionophores were added determined which metal ions entered the GUVs and to what extent, and the prior addition of one ionophore inhibited transport with subsequent ionophores in all three cases.

These observations raise the question of what the mechanism is for the negative feedback of the ionophores among each other. In further experiments, we observed that, when the GUVs were first incubated with one of the metal ions (Ni^2+^, Cu^2+^ or Ca^2+^) and then a cocktail of the three ionophores was added at once, none of the metal ions was transferred into the GUVs (Supplementary Fig. 14a–c). This observation indicates that the insertion of spectator ionophores decreases the ability of the cognate ionophore to transport its metal ion across the membrane and that there is no kinetic preference of one ionophore over the other inserting faster into the GUVs. Moreover, we investigated the effect of different concentrations of the first ionophore on transport with subsequent ionophores (Fig. 4g and Supplementary Figs. 15 and 16). In each case, we observed that, when the first ionophore was added at working concentration (20 µM, 5 µM and 1 µM for ionophores A, B and C, respectively), the transport with the second ionophore decreased drastically. These results suggest that the functionality of the second ionophore in the GUV membrane depends closely on the amount of the other ionophores present in the membrane.

### MD simulations provide microscopic dynamic insight

**Fig. 5:**
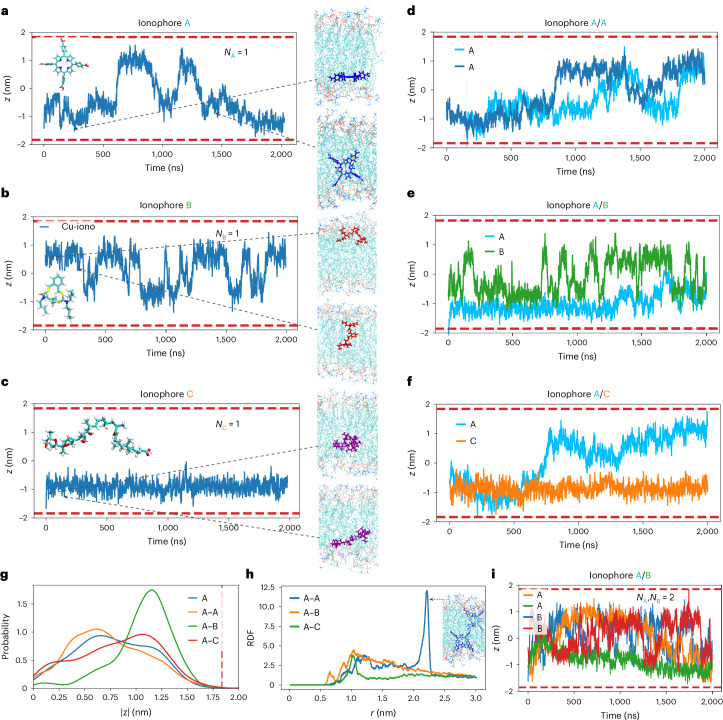
MD simulation of ionophores in a POPC bilayer. **a**–**c**, The time-dependent position along the membrane normal (*z* coordinate) of the COM of a single ionophore for the three ionophores A (**a**), B (**b**) and C (**c**), respectively. The position of the centre of the membrane is at *z* = 0, and the approximate average positions of the lipid head group in the two leaflets are represented by dashed red lines. On the right side are some snapshots. **d**, Similar to **a** but with *N*_A_ = 2. **e**, Similar to **a** but with *N*_A_ = *N*_B_ = 1. **f**, Similar to **a** but with *N*_A_ = *N*_C_ = 1. In **d**–**f**, the traces of ionophores A, B and C are shown in (light and dark) blue, green and orange, respectively, and each ionophore is shown individually. **g**, The probability of finding ionophore A at a specific *z* value in the intensity-sensitive representation (Methods). For the case of two ionophores, only those snapshots where both ionophores are in the same leaflet contribute. **h**, The RDFs for the compositions, shown in **d**–**f**. A snapshot is shown for the specific configuration of ionophore A corresponding to the peak at ~2.2 nm for A–A pairs. **i**, Similar to **a** but with *N*_A_ = *N*_B_ = 2.

To gain a better molecular picture of the interactions between the ionophores inside the GUV membrane and understand how they negatively influence each other’s activity, we conducted molecular dynamics (MD) simulations for all three ionophores in a POPC bilayer. In these simulations, we considered the comparatively rigid and planar ionophore A (C_48_H_30_N_4_O_8_, C_4_ symmetric), the more flexible ionophore B (C_26_H_44_N_2_S_4_, C_2_ symmetric) and the larger, flexible and symmetry-free ionophore C (C_41_H_72_O_9_). As can be seen in the time evolution of the *z* coordinate of the centre of mass (COM) of all three ionophores within the membrane, ionophores A and B switched several times between the two leaflets within the 2,000 ns simulation time, albeit with many more transitions for ionophore B than ionophore A (Fig. 5a,b). Interestingly, the orientation of ionophore A was parallel to the membrane surface when close to the head group and otherwise orthogonal inside the lipid bilayer, presumably allowing the transport through the membrane, but no such preferred orientation was seen for ionophore B (Supplementary Fig. 17). Furthermore, no transitions were observed for ionophore C within the same simulation time of 2,000 ns (Fig. 5c). Transitions of ionophore C may happen at longer time scales, but these become too demanding in terms of computational time. However, one may investigate the impact of ionophore C on the other species within the lipid bilayer in these simulations.

Additional information is gained in considering the mutual impact of ionophores on each other. For this purpose, trajectories of systems with two ionophores were considered, namely, the combination of ionophore A with any of the ionophores A, B or C, involving interactions between like and unlike ionophores (Fig. 5d,f). This corresponds to two-dimensional densities of 0.05 ionophores nm^−2^ (2 ionophores in 106 POPC lipids). To improve the statistical analysis, we generated each of the trajectories with unlike ionophores twice in independent simulations. The number of transitions between the two leaflets of the membrane for ionophore A was 4 for single ionophore A (*N*_A_ = 1), 3 in the presence of a second ionophore A (*N*_A_ = 2), 0.5 in the presence of one ionophore B (*N*_A_ = *N*_B_ = 1) and 0.5 in the presence of one ionophore C (*N*_A_ = *N*_C_ = 1), using the average over both trajectories for the final two cases. Thus, there were hardly any transitions of ionophore A when a second unlike ionophore was present, and this effect was weaker for the addition of a like ionophore. On closer inspection, it became evident that ionophore A was shifted to the outer part of the membrane when a second unlike ionophore was present as compared with the case of a single ionophore A (Fig. 5e,f). This shift is probably a direct consequence of the interaction among the ionophores and naturally explains the emergence of a reduced transition rate of ionophore A. A more quantitative account of this observation is shown in Fig. 5g, where the position of ionophore A is clearly shifted to the periphery when ionophore B or C is present in the same leaflet of the membrane. In contrast, the presence of a second ionophore A had no noticeable impact on ionophore A’s position in the membrane, which is in agreement with the remaining ability to transition between the different leaflets of the membrane.

For all three pairwise interactions (A–A, A–B and A–C), the radial distribution function (RDF) displayed considerable deviations from unity around 1 nm, consistent with interactions among the ionophores within the same leaflet (Fig. 5h). However, the differences are not as pronounced and much longer simulations would be required, involving an averaging over different interaction patterns, to obtain fully convergent behaviour of the RDF. There were only weak interactions beyond 1 nm except for the peak at ~2.2 nm for A–A pairs, which corresponds to a specific edge-to-edge binding where each ionophore A is in a different leaflet (Fig. 5h).

The corresponding pairwise interactions involving ionophores B and C were also considered (Supplementary Fig. 18). Despite the important deviations in the RDF from unity for the B–B and B–C pairs (Supplementary Fig. 19), the number of transitions of ionophore B was not notably perturbed by the presence of a second ionophore (16 transitions for *N*_B_ = 1, 12 transitions for *N*_B_ = 2, 17 transitions for *N*_B_ = *N*_A_ = 1 and 13 transitions for *N*_B_ = *N*_C_ = 1). However, there was a substantial inward shift of ionophore B in the presence of ionophore C (Supplementary Fig. 20a), which may reduce the binding probability to a metal ion at the surface.

Further information about the influence of ionophore density in the membrane on ionophore mobility can be gained from studying systems with more than two ionophores. Indeed, at higher densities of ionophores A (*N*_A_ = 4), transitions between the leaflets were no longer possible (Supplementary Fig. 21a) due to considerable clustering among the ionophores (Supplementary Fig. 22). Similarly, clustering effects were observed for ionophore B (Supplementary Fig. 23), although, in contrast to ionophore A, they were only in the form of transient loosely packed structures. Nevertheless, for *N*_B_ = 4, the number of transitions reduced to 7 compared with the 16 transitions for *N*_B_ = 1 (Supplementary Fig. 21b). Lastly, in systems that included a mixture of multiple ionophores, that is, *N*_A_ = *N*_B_ = 2, the switching between the two leaflets for ionophore A was also strongly reduced (one transition compared with three for *N*_A_ = 2 and four for *N*_A_ = 1) (Fig. 5i), due to expected interactions between ionophores A and B.

Overall, the MD simulations of ionophore A with either ionophore A, B or C indicate noticeable interactions among the ionophores, which impact the transition rate between the two leaflets of the membrane and are likely to give rise to less effective metal ion transport. Effects are also seen for ionophore B with either ionophore B or C. Moreover, the identified interactions between the different ionophores and their tendency to form clusters within the membrane are a possible molecular origin of the negative effect that the ionophores have on each other’s ability to transport metal ions across the membrane.

### Ionophore sequence determines fate of synthetic cells

In pluripotent cells, the commitment towards one fate means gaining new capabilities while simultaneously losing other dormant functions. The pluripotent cell makes these decisions on the basis of the spatiotemporal context of different cues in its environment and differentially up- or downregulates functions accordingly. Moreover, the process of differentiation can go through multiple steps, in which the cell first converts to a more specified multipotent cell and in a second step reaches a terminally differentiated state. Analogously, we envisioned that memory of the GUVs for the sequence in which the ionophores were added provides a basis to build pluripotent synthetic cells, which can be differentiated in multiple steps. In other words, the first ionophore would lead to commitment to a certain fate (fate A, increased internal pH; fate B, H_2_O_2_ production; or fate C, lysis) and convert the pluripotent into a multipotent GUV. Subsequently, the second ionophore can terminally differentiate the GUV by relying on the residual activity of the second ionophore.

To test this multistep differentiation towards different cell fates, we placed the above-described pluripotent GUVs (membrane shown in red) with the co-encapsulated apo-enzymes (apo-urease, apo-GaoA and apo-PLA_2_), substrates and urease and GaoA sensors (shown in cyan and green, respectively) or the membrane-impermeable dye, sulfo-Cy5 (shown in orange), into environments with external Ni^2+^, Cu^2+^ and Ca^2+^ ions.

**Fig. 6:**
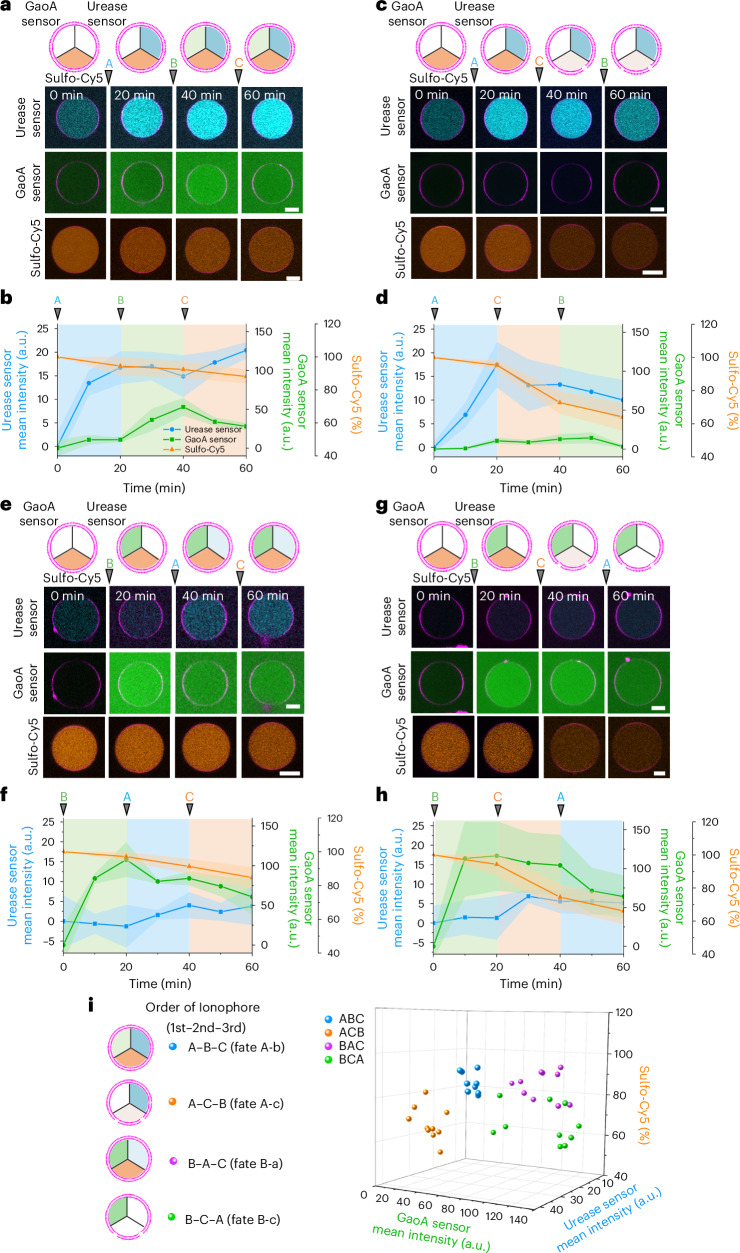
Differential activation of pluripotent GUV through sequential addition of ionophores. **a**,**c**,**e**,**g**, Representative CLSM images of GUVs (membrane shown in red) loaded with three apo-metalloenzymes in the presence of all three metal ions as different ionophores were added every 20 min. Order of ionophores is A-B-C (**a**), A-C-B (**c**), B-A-C (**e**) and B-C-A (**g**). The GUVs were loaded either with urease sensor (shown in cyan) and GaoA sensor (shown in green) or sulfo-Cy5 (shown in orange) for monitoring permeability. **b**,**d**,**f**,**h**, The mean fluorescence intensity of urease sensor (blue, *n*_GUV_ = 10) and GaoA sensor (green, *n*_GUV_ = 10), or sulfo-Cy5 (orange, *n*_GUV_ = 10) inside GUVs in **a** (**b**), **c** (**d**), **e** (**f**) and **g** (**h**). **i**, Three-dimensional plots showing final states of GUVs exposed to the three ionophores in different orders (*n*_GUV_ = 10). Individual GUVs either loaded with the urease and GaoA sensors or with sulfo-Cy5 were tracked throughout the experiment, and each GUV is from an independent sample. The error bars with fill area indicate the s.d. of the average intensities for *n*_GUV_ = 10. Scale bars, 10 µm. Source data

Upon addition of ionophore A, the signal from the urease activity sensor increased over the course of 20 min owing to the increase in internal pH, that is, fate A (Fig. 6a–d). Depending on which ionophore was added second, the subsequent response of the GUVs differed. If ionophore B was added second, the signal from the GaoA sensor increased slightly, showing low levels of H_2_O_2_ generation, and this was classified as fate A-b (Fig. 6a,b). When ionophore C was added to these GUVs as the third ionophore, there was no further change, representing a terminally differentiated GUV that is unresponsive to further inputs. In contrast, if ionophore C was added second, there was no response from the GaoA sensor and the GUVs did not lyse, but they became leaky due to partial PLA_2_ activation as observed through the release of loaded sulfo-Cy5 (shown in orange), resulting in fate A-c (Fig. 6c,d). Subsequently, the addition of ionophore B as the third one did not activate GaoA activity.

The outcome for the pluripotent GUV was different if ionophore B was added first instead of ionophore A. In this case, the signal from the GaoA sensor increased rapidly due to the transport of Cu^2+^ into the GUV and the activation of GaoA, resulting in fate B (Fig. 6e–h). If ionophore A was added second 20 min later, there was almost no increase in the urease sensor, fate B-a (Fig. 6e,f). Not observing an increase in pH may also be partially due to the further oxidation of d-galacto-hexodialdose to the corresponding acid with H_2_O_2_. Moreover, in this case, the addition of ionophore C as the third one had no consequence for GUV stability or membrane permeability. In contrast, if ionophore C was added second after ionophore B, the GUVs got leaky due to residual PLA_2_ activation, resulting in fate B-c (Fig. 6g,h).

The addition of ionophore C first leads to the lysis of the GUVs (fate C), as already demonstrated (Fig. 3f), but its addition as the second ionophore activates only low levels of PLA_2_. Consequently, the GUVs remain stable but become somewhat leaky (Fig. 6d,h and Supplementary Fig. 24). Finally, when ionophore C was added third, this resulted in insufficient Ca^2+^ influx into the GUVs and the GUVs remained intact and tight. When we increased the concentration of encapsulated apo-PLA_2_ to 500 nM and repeated the sequential addition of ionophores, we observed more prominent leakage of sulfo-Cy5 when ionophore C was added second (60% over 20 min) and minor leakage sulfo-Cy5 when ionophore C was added third (20% over 20 min). Most importantly, also in this case, only the addition of ionophore C first resulted in lysis (Supplementary Fig. 24). A three-dimensional plot of the final mean fluorescence intensities in the three channels delineates the four different outcomes of enzyme activation depending on the sequence in which the ionophores were added, with some overlap in fate B-a and fate B-c (Fig. 6i and Supplementary Figs. 25–27). Overall, the same pluripotent GUV was able to adapt five different fates, depending on the order in which the three ionophores were added, each step resulting in desensitization towards subsequent ones.

## Conclusion

We have demonstrated the selective activation of three different apo-metalloenzymes in a GUV through the selective transport of specific metal ions via their ionophore. The GUV displayed intracellular pH increase, H_2_O_2_ generation or cell lysis in response to addition of ionophore A, B or C, respectively. As an important property of the GUV, metal ion transport depends on the order in which the ionophores are added due to favoured ionophore interactions in the membrane, revealed by MD simulations, reducing their ability to move across the membrane and transport metal ions. This property allowed the pluripotent GUV to differentiate towards different fates: The first added ionophore determines differentiation into one of the cell types: intracellular pH increase (fate A), H_2_O_2_ generation (fate B) or cell lysis (fate C) as in a pluripotent cell. The resulting multipotent GUVs can further differentiate towards one of two subfates upon addition of a second ionophore (fate A-b, fate A-c, fate B-a or fate B-c), but the addition of a third ionophore has no further effect, reminiscent of a completely differentiated cell with a third dormant function that can no longer be activated.

Changes in cellular phenotype can be regulated at the level of transcription/translation and at the posttranslational level through protein modifications and effector molecules. In the context of differentiation in synthetic cells, the work presented here is distinct from previous examples of synthetic cell differentiation that mostly rely on altered gene expression for gaining new enzymatic activity^13,22^, in that it regulates enzymatic activity in a lipid-bound synthetic cell at the posttranslational level, allowing more direct and faster regulation of the cellular phenotype than in transcriptional regulation. The differential activation of the enzymes depends on specific ion transporters, that is, ionophores, a feature not achievable with commonly used pore-forming proteins until now. Furthermore, the unexpected cross-talk among the different ionophores results in distinct phenotypes, depending on the sequence in which they are added, which was not reported in other studies of synthetic cell differentiation.

The adaptive metal ion transport described herein provides the basis of different fate choices without being genetically coded. About one-third of all human proteins are estimated to require metal ions for proper function, and metalloenzymes are important in all parts of metabolism^42^. Thus, the posttranslational regulation of metalloenzymes through metal availability provides a faster alternative than in vitro transcription and translation commonly used to steer differentiation in synthetic cells^13,17,22,24^. Moreover, the high specificity that different metalloenzymes have for their cofactors and the large repertoire of selective ionophores make it possible to steer distinct enzymatic pathways. Currently, such high specificity is enabled only by DNA-based reaction networks, which are difficult to link with metabolic activity^19,25^. In fact, various metalloenzymes are already implemented in synthetic cells for signal transduction, cell division or cell lysis and could be triggered by using ionophore-mediated metal ion transport^6,40,43,44^. Here, we chose only three metalloenzymes with Ni^2+^, Cu^2+^ and Ca^2+^ as cofactors as demonstrators, but metalloenzymes that require different metal ion cofactors (for example Mn, Fe, Zn, Co or Mo) or are activated at different metal ion concentration ranges equally open possibilities for metalloregulation in synthetic cells^45,46^. Metalloregulation may also lead to applications in biomedicine and bioremediation considering that some of these metal ions are important secondary messengers in biology or heavy metal toxins.

## Methods

### GUV preparation and washing

GUVs were prepared by the polyvinylalcohol (PVA)-gel assisted formation method^40,47^. In detail, a PVA solution was prepared by mixing 5% (w/v) PVA (molecular weight 145,000 g mol^−1^) in Milli-Q water with 100 mM sucrose overnight at 565*g* at 80 °C. Forty microlitres of the PVA solution was dried as a thin film on a glass slide (60 × 24 mm) at 50 °C for 30 min. Then, 5 µl of a lipid 10 mg ml^−1^ POPC with 0.2 mol% DiD dye was spread on the PVA layer and dried for 1 h at 30 °C. Using a Teflon chamber (ca. 40 mm × 24 mm) as a spacer and a second glass slide, a chamber was built on top of the slide with PVA and lipid layers. Then, the lipids were hydrated with 1 ml of 100 mM sucrose in buffer A (50 mM Tris and 100 mM NaCl, pH 7.4) containing all the components to be loaded into the GUV depending on the experiment (2 µM Rhod2, 100 µM CaCl_2_, 1 µM apo-urease, 150 nM apo-GaoA, 80 nM apo-PLA_2_ (80 nM PLA_2_ with 30 µM EDTA, if not specified otherwise), 100 mU HRP, 1 mM galactose and 10 µM urease sensor) for 1 h at room temperature allowing GUV formation. After that, the chamber was inverted for 5 min and gently taped twice using a pipette tip, and the GUVs were collected into a 1.5 ml LoBind Eppendorf tube.

Next to wash away excess loaded components, 1 ml buffer A supplemented with 100 mM glucose was added to the GUVs, and the GUVs were allowed to settle overnight at 4 °C in the dark. The next day, the top 1 ml of the buffer was removed without disturbing the bottom layer. The GUVs were washed a second and third time as before, but the GUVs were only allowed to settle for 2 h at room temperature in dark. The bottom 100 µl of GUV preparation was used in further experiments. In all experiments, 100 µl of buffer A (100 mM glucose) was mixed with 100 µl of GUV solution in 18-well chambers precoated with 3% bovine serum albumin.

### Transport of metal ions into Rhod2-loaded GUVs

GUVs were loaded with 2 µM Rhod2 and only if the transport of Ni^2+^ or Cu^+2^ was analysed with additional 100 µM CaCl_2_. The GUVs were mixed with 1 µM NiCl_2_, 1 µM CuCl_2_ or 100 µM CaCl_2_ in µ-slide 18-well glass-bottom chambers, and the Rhod2 fluorescence in the GUVs was monitored for 20 min before adding the ionophores (20 µM ionophore A, 5 µM ionophore B and 1 µM ionophore C if not specified otherwise). In experiments where the GUVs were exposed to ionophore C, 100 µM CaCl_2_ was added to the outer solution to avoid diffusion of the intracellular Ca^2+^ to the surrounding medium. To check the ion transport efficiency according to the ionophore sequence, each ionophore was added at 20 min intervals. The process at each step was monitored by acquiring images in the DiD and Rhod2 channels using confocal microscopy.

The percentage of transport for Ni^2+^ and Cu^2+^ in each GUV loaded with [Ca^2+^-Rhod2] was calculated as follows: percentage of transport = (*I*_initial_ − *I*_final_)/*I*_mean_ × 100, where *I*_initial_ and *I*_final_ are the Rhod2 fluorescent intensity in a GUV before and after addition of ionophore and *I*_mean_ is the initial mean Rhod2 fluorescence intensity of all GUVs analysed in the sample (*n*_GUV_ = 10–50).

The relative transport percentage for Ca^2+^ in each GUV loaded with Rhod2 was calculated as follows: percentage of relative transport = (*I*_final_ − *I*_initial_)/average (*I*_final (ionophore C + Ca_^2+^_)_ − *I*_initial (ionophore C + Ca_^2+^_)_) × 100. Here, *I*_initial_ and *I*_final_ represent the Rhod2 fluorescent intensity in a GUV before and after the addition of ionophore, respectively. The average of (*I*_final (ionophore C + Ca_^2+^_)_ − *I*_initial (ionophore C + Ca_^2+^_)_) across samples is considered as 100% transport (*n*_GUV_ = 10–50).

### Measurement of transport efficiency of each ionophore using a plate reader

When the transport of Ni^2+^ or Cu^+2^ was analysed, GUVs were loaded with 2 µM Rhod2 and 100 µM CaCl_2_. Two-hundred microlitres of GUVs were mixed with 1 µM NiCl_2_ or 1 µM CuCl_2_ in a 96-well transparent bottom plate, and the Rhod2 fluorescence in the GUVs was monitored for 30 min with a multimode plate reader (Tecan Spark) before adding 0–20 µM ionophore A or ionophore B, respectively. In experiments where the GUVs were exposed to ionophore C, GUVs were loaded with only 100 µM CaCl_2_. Two-hundred microlitres of GUVs were mixed with 2 µM Rhod2 in a 96-well transparent bottom plate, and the Rhod2 fluorescence in the GUVs was monitored for 30 min after adding 0–2 µM ionophore C. It should be noted that, unlike in the experiments where GUVs were monitored with confocal microcopy, for ionophore C in the plate reader the GUVs were loaded with CaCl_2_ and the Rhod2 dye was added outside the GUVs. Moreover, the yield of the GUVs and the washing efficiency in each preparation may vary. Therefore, the values were normalized within each set of experiments when calculating the percentage of transport. All experiments were performed in triplicate, and the error bars represent the standard deviation (s.d.). The percentage of transport for Ni^2+^/ionophore A and Cu^2+^/ionophore B was calculated as follows: percentage of transport = (*I*_initial_ − *I*_*x*_)/(*I*_initial_ − *I*_min_) × 100; and for Ca^2+^/ionophore C was calculated as follows: percentage of transport = (*I*_*x*_ − *I*_initial_)/(*I*_max_ − *I*_initial_) × 100, where *I*_initial_ and *I*_*x*_ are the Rhod2 fluorescence intensity at time zero and at any given timepoint, respectively, and *I*_min_ and *I*_max_ the minimum and maximum Rhod2 fluorescence intensity measured within one set of experiments with different ionophore concentrations.

### Preparation of apo-urease

To remove Ni^2+^ from holo-urease, 100 µM urease in 500 µl buffer A was mixed with 5 µl 500 mM dimethylglyoxime (DMG) in 50 mM Tris–HCl, pH 8.0 buffer containing of 1% ethanol and 100 mM NaCl overnight at room temperature. As the DMG–Ni complex precipitated, the sample was centrifuged at 14,000*g* for 3 min and the supernatant was mixed once more with 5 µl 500 mM DMG solution overnight at room temperature^48^. After the centrifugation, the resultant supernatant was used as apo-urease. To access the catalytic activity of urease, 10 µM fluorescein (urease sensor, pH-dependent fluorescence), 1 µM apo-urease and 1 µM NiCl_2_ were mixed in 200 µl buffer A, and the fluorescence of the fluorescein (*λ*_ex_ = 498 nm, *λ*_em_ = 517 nm) was measured in regular intervals upon addition of 10 mM urea with a multimode plate reader (Tecan Spark). The holo-urease and apo-urease were used as positive and negative controls, respectively.

### Preparation of apo-GaoA

The plasmid coding for GaoA was transformed into *Escherichia coli* BL21 (DE3), and a starter culture was grown from a single colony in 10 ml lysogeny broth (LB) medium with 50 μg ml^−1^ ampicillin at 37 °C at 280*g* overnight. The culture was centrifuged (8,500*g*, 4 °C, 10 min), and the obtained pellet was resuspended in 1 l M9 salts supplemented with 0.4% glucose, 1 mM MgSO_4_, 0.3 mM CaCl_2_ and 50 μg ml^−1^ ampicillin. The culture was grown at 37 °C at 280*g*until an OD_600_of 0.4–0.6. At that point, protein expression was induced with 0.5 mM isopropyl β-d-1-thiogalactopyranoside and the culture was incubated at 16 °C overnight at 280*g*. The bacteria were collected by centrifugation, resuspended in 20 mM sodium phosphate buffer pH 7.4 containing 500 mM NaCl, 1 mM PMSF and 30 mM imidazole and lysed by sonication. The lysate was cleared by centrifugation (17,000*g*, 1 h, 4 °C), and the supernatant was filtered through 0.2 µm polyethersulfone membrane. The His-tagged GaoA was purified with Ni-affinity chromatography (5 ml HiTrap chelating HP column, GE Healthcare) using a 30 to 400 mM step gradient of imidazole (5 column volumes (CVs) 30 mM imidazole, 2 CVs 100 mM imidazole, 4 CVs 200 mM imidazole and 2 CVs 400 mM imidazole) following previously described protocols^46^. The collected fractions were analysed by SDS–PAGE (Supplementary Fig. 28), and the GaoA was purified in the apo-form as confirmed with the activity assay. To access the catalytic activity of GaoA, the Amplex red assay was used. For this purpose, 150 nM apo-GaoA, 0.12 nM HRP and 1 mM galactose were mixed in 200 µl buffer A and the fluorescence of the fluorescent product resorufin (*λ*_ex_ = 571 nm, λ_em_ = 584 nm) was measured in regular intervals upon addition of 50 µM Amplex red with a multimode plate reader (Tecan Spark). To activate the apo-GaoA, 750 nM CuCl_2_ was added into the solution just before initiating the measurements.

### apo-Enzyme activation in GUVs

GUVs were prepared with either a single apo-metalloenzyme with its respective substrate and sensors (1 µM apo-urease with 10 µM urease sensor; 150 nM apo-GaoA with 1 mM galactose and 100 mU HRP; and 80 nM PLA_2_ with 30 µM EDTA) or three apo-metalloenzymes with all substrates and sensors. Then, 10 mM urea and 50 µM Amplex red as GaoA sensor were added to the surrounding medium where appropriate. In all experiments, single or three metal ions (1 µM NiCl_2_, 1 µM CuCl_2_ and/or 100 µM CaCl_2_) were first added and incubated with the GUVs for 20 min before adding the first ionophores. For the activation of single apo-enzymes, also the addition of the ionophore first and the metal ion after 20 min was evaluated. Then, images of the GUVs were acquired in relevant channels and images were acquired in regular intervals. Subsequent ionophores were added with 20 min intervals, and the sensor response was measured at defined timepoints. All images were analysed using ImageJ 1.52b, and if not specified that the same GUV was tracked, randomly selected GUVs in the field of view were analysed. It should be noted that the addition of metal ions and ionophores leads to the movement of GUVs in the sample and the GUVs analysed at different timepoints may not be the identical GUVs.

For the analysis of the permeability due to a partial activation of apo-PLA_2_, the fluorescence intensity of 2 µM sulfo-Cy5 loaded with 80 nM or 500 nM apo-PLA_2_ and 30 µM EDTA into GUVs (membrane labeled with 1,1′-dioctadecyl-3,3,3′,3′-tetramethylindocarbocyanine perchlorate (DiI)) was measured for least ten randomly selected GUVs before and 20 min after addition of ionophore.

For the analysis of urease and GaoA activity, the mean intensity inside the GUVs (*n*_GUV_ = 10) was measured over time in the same sample after adding the NiCl_2_ or CuCl_2_ and then ionophore A or B or the ionophores first and the metal ions second.

For the analysis of PLA_2_ activity, GUVs areas were measured over time in the same sample after first adding the CaCl_2_ and then ionophore C (*n*_GUV_ = 3) or after first adding ionophore C and then CaCl_2_ (*n*_GUV_ = 6). To analyse the area of GUVs, raw microscopy images (8 bit) in the DiD channel were opened in ImageJ, separating the different fluorescence channels. Single GUVs were defined through their membrane dye (DiD or DiI staining). The ‘default’ thresholding method (accessible under Image > Adjust > Threshold) was used, with adjustments made to the threshold settings as needed to highlight the GUV membranes against the background. Following the application of the appropriate threshold, the ROI Manager (accessible via Analyze > Tools > ROI Manager) was then utilized to create and select regions of interest (ROIs). Specifically, ‘oval selection’ was utilized for the circular membrane of GUVs before lysis, while ‘freehand selection’ was used for the GUV membrane at the onset of lysing, forming a micelle structure. Subsequently, the selected GUV ROIs were added to the ROI Manager window, overlaying them onto the image. Upon clicking the ‘Show All’ and ‘Measure’ buttons, the selected ROIs were displayed overlaid on the image, with area measurements expressed in square micrometres.

To analyse the changes in the permeability of GUVs resulting from the partial activation of apo-PLA_2_, GUVs were loaded with 80 nM or 500 nM apo-PLA_2_, 30 µM EDTA and 2 µM sulfo-Cy5. Subsequently, changes in the fluorescence intensity of the loaded sulfo-Cy5 were measured on ten randomly selected GUVs after the addition of ionophore C at 20, 40 and 60 min. The percentage of sulfo-Cy5 within each GUV was computed as follows: % Sulfo-Cy5 = *I*_20 min/40 min/60 min_/(*I*_0min_) × 100. Here, the initial intensity value (*I*_0min_) at 0 min is considered as the baseline, set to 100%. The fluorescence intensity at 20 min, 40 min and 60 minutes (*I*_20min_, *I*_40min_ and *I*_60min_, respectively) is normalized against the baseline intensity. This normalization process yields the percentage of sulfo-Cy5 present within the GUV at the specified timepoints.

### Chemiluminescence inside the GUVs

GUVs containing apo-GaoA, galactose and HRP were prepared without membrane dye as described above and washed three times. Two-hundred microlitres of settled GUVs from the bottom of the tube or exterior buffer from the top of the tube were added into a 96-well white bottom plate. The chemiluminescence from the GUVs was measured with a plate reader (Tecan Spark) in the presence of 50 µM luminol and 1 µM external CuCl_2_ upon addition of 10 µM ionophore B.

### Confocal fluorescence microscopy

All images were acquired on a Leica SP8 confocal laser scanning microscope (CLSM) through a 63× water objective. The urease sensor (*λ*_ex_ = 460 nm, *λ*_em_ = 510 nm) and DiO (*λ*_ex_ = 484 nm, *λ*_em_ = 501 nm) were excited with a 488 nm laser and emission was detected at 505–559 nm and 493 − 581 nm, respectively. Rhod2 (*λ*_ex_ = 552 nm, *λ*_em_ = 581 nm) and GaoA sensor (resorufin; *λ*_ex_ = 571 nm, *λ*_em_ = 584 nm) were excited with a 552 nm laser and emission was detected at 557–604 nm and 575–628, respectively. DiD (*λ*_ex_ = 644 nm, *λ*_em_ = 665 nm) and sulfo-Cy5 (*λ*_ex_ = 646 nm, *λ*_em_ = 662 nm) were excited with a 638 nm laser, and the emission was detected at 657–691 nm and 652–703 nm, respectively.

### System preparation for MD simulations

Force field parameters for the ionophores A, B and C were obtained from the general Amber force field^49^. To estimate the partial charges, first the geometry of the molecules was optimized on the B3LYP/6-31G* (ref. ^50^) level of theory employing Gaussian16^51^. Afterwards, the restrained electrostatic potential charges were calculated by Antechamber^52^ from the electrostatic potential (ESP) charges obtained from a Hartree-Fock/6-31G* calculation^50^.

Next, a pure POPC membrane was prepared using CHARMM-GUI membrane builder^53^. For each ionophore, first one ionophore was inserted at a distance of 3 nm from the centre of the membrane, and subsequently, water molecules were added to the system using GROMACS tools. The simulation was run until the ionophore enters the membrane. Then, a second ionophore was added to the system and the simulation were run until it is absorbed to the membrane. This process was repeated until the different required number of ionophores were inside the membrane. In case of simulations of systems with two unlike ionophores, for each combination two samples were simulated starting with a configuration in which the two ionophores are in the same or in the opposite leaflet. For the case of ionophore C, the ionophores did not enter the membrane on the MD time scales of several hundred nanoseconds. Thus, we additionally used a harmonic bias constant with a force constant of 1,500 kJ mol^−1^ nm^−2^. A rate of 1 ns nm^−1^ was applied to force the ionophore to enter the membrane. The force was removed once the ionophores were in the membrane. All simulations were conducted for 2 μs with a time step of 2 fs. We never observed an ionophore leave the membrane.

### MD simulations

The MD simulations of the ionophore–membrane systems were performed using version 2019.6 of GROMACS^54^, and the structure and parameter files were obtained as described above. The TIP3 model was used for the water molecules. Periodic boundary conditions were applied in all directions. The long-range electrostatic interactions were treated using particle mesh Ewald^55^, with a cut-off distance of 1.2 nm and compressibility of 4.5 × 10^−5^.

To treat the van der Waals (vdW) interactions, cut-off schemes with a cut-off distance of 1.2 nm were used, smoothly truncated between 1.0 and 1.2 nm. Constant pressure at 1 bar was controlled by using the Berendsen^56^ and the Parrinello–Rahman^57^ barostat in equilibration and production simulations, respectively, with the semi-isotropic pressure scheme. To control the temperature at 310 K the system was coupled to the Nosé–Hoover thermostat^58^. To constrain bonds, the LINCS algorithm was used^59^. All systems were first minimized using the steepest descent algorithm and sequentially equilibrated using first the NVT and then the NPT protocol in multiple steps by restraining the lipids as it is set by CHARMM-GUI membrane builder.

The simulation data were analysed using GROMACS tools and in-house codes in Python, incorporating MDAnalysis^60,61^. VMD (visual molecular dynamics) was used to visualize the trajectories and to prepare the snapshots^62^.

### Coordinate system

The *z* axis is defined as the membrane normal. *z* = 0 corresponds to the centre of the membrane, defined as the COM of the P atoms of lipids.

### Identification of transition events

To identify transitions of the ionophores between the two leaflets, we imposed the condition that before and after the transition event the *z* value (position of the COM along the membrane normal) must reach a certain distance from the centre of the membrane (of course, one with a positive and one with a negative sign). For ionophore A, this distance is chosen to be 0.7 nm, for ionophore B 0.6 nm. Both values are close to the typical distance of the ionophore from the centre of the membrane. In this way, it is ensured that all transitions reflect a full transfer from one leaflet to the other. Since ionophore C shows no transitions except in one case, no quantitative criterion was needed.

### Height distributions

We used two different types of height distribution. For individual ionophores we determined the histogram of *z* values. Typically, the histogram involves usually two peaks if the ionophore visits the two leaflets, one for positive *z*and one for negative *z* values, but with different heights, depending on the localization of the specific ionophore. For the intensity-sensitive representation, one chooses the peak with the higher maximum. If this corresponds to positive (negative)*z*values, only the data for positive (negative) *z* values are kept. In this way, one keeps information about the statistical relevant behaviour. Then, in a second histogram, these data are stored as a function of |*z*|. Furthermore, when two ionophores are present, for some analyses we restricted ourselves to the configurations where both ionophores are in the same leaflet (explicitly mentioned in the text, if applicable). All relevant simulation data are accessible from the authors on reasonable request.

### Data analysis

The data are presented as the mean ± s.d. from at least ten GUVs in observed with confocal microscopy. In plate reader experiments, measurements were performed in independent triplicates.

## Online content

Any methods, additional references, Nature Portfolio reporting summaries, source data, extended data, supplementary information, acknowledgements, peer review information; details of author contributions and competing interests; and statements of data and code availability are available at 10.1038/s41557-024-01682-y.

## Supplementary information

Supplementary Information Supplementary Figs. 1–28.

Supplementary Data 1 Statistical data.

Supplementary Movie 1 GUV loaded with apo-urease after addition of ionophore A in the presence of external NiCl_2_ deform in some instances. The membrane is shown in purple.

Supplementary Movie 2 GUVs loaded with apo-PLA2 after addition of ionophore C in the presence of external CaCl_2_ undergo lysis. The membrane is shown in purple.

Supplementary Movie 3 GUV loaded with the three apo-enzymes undergo lysis after addition of ionophore C in the presence of all three metal ions. The membrane is shown in purple, and the urease sensor is shown in cyan.

## Source data

Source Data Fig. 1 Statistical source data.

Source Data Fig. 2 Statistical source data.

Source Data Fig. 3 Statistical source data.

Source Data Fig. 4 Statistical source data.

Source Data Fig. 6 Statistical source data.

## Data Availability

All relevant data supporting the findings of this study are available within the paper and the Supplementary Information. Source data are provided with this paper.
