# Supplementary material for: Adaptive metal ion transport and metalloregulation-driven differentiation in pluripotent synthetic cells
Source: Nat Chem. 2024 Dec 23;17(1):54–65. doi: 10.1038/s41557-024-01682-y (PMC11703756; doi:10.1038/s41557-024-01682-y)
Supplement: Supplementary file 1 — Supplementary Figs. 1–28. [file 41557_2024_1682_MOESM1_ESM.pdf]

nature chemistry

Supplementary information https://doi.org/10.1038/s41557-024-01682-y

Adaptive metal ion transport and metalloregulation-driven differentiation in pluripotent synthetic cells

In the format provided by the authors and unedited

Table of content

1. Materials **2**

2. Supplementary Figures (Figure S1-S28) **3–30**

Materials.

Lipids– 1-Palmitoyl-2-oleoylphosphatidylcholine (16:0-18:1 PC) (Cat# 850457) was purchased from Avanti Polar Lipids in chloroform. The membrane dye 1,1'-Dioctadecyl-3,3,3',3'-Tetramethylindodicarbocyanine (DiD, Cat# D7757-10 mg) and the calcium sensitive dye Rhod2 (Cat# R14220) were purchased from Thermo-Fisher Scientific.

Ionophores – ionophore A (meso-Tetraphenylporphine-4,4′,4″,4‴-tetracarboxylic acid 4,4′,4″,4‴-(Porphine-5,10,15,20-tetrayl)tetrakis(benzoic acid), Cat# 42156); ionophore B (o-Xylylenebis(N,N-diisobutyldithiocarbamate), Cat# 61193); ionophore C (Ionomycin from *Streptomyces conglobatus,* Cat# I9657) were purchased from Sigma-Aldrich.

Enzymes – PLA_2 (Phospholipase A2 from honeybee venom *Apis mellifera*, Cat# P9279); Urease from *Canavalia ensiformis* (Cat# U1500); HRP (Peroxidase from horseradish, Cat# P6782) were purchased from Sigma-Aldrich. The plasmid DNA coding for galactose oxidase (GaoA) was kindly provided by Prof. Nicholas J. Turner (the Manchester Institute of Biotechnology, University of Manchester).[47]

Dimethylglyoxime disodium salt octahydrate (DMG, Cat# 10209700) was purchased from Thermo Scientific. All others chemicals including 8-hydroxy-pyrene-1,3,6-trisulfonic acid trisodium salt (urease sensor, Cat# H1529); fluorescein (Cat# 46955); Amplex Red (Cat# 90101); luminol (Cat# 123072); EDTA (Cat# E5134); urea (Cat# U5378); galactose (Cat# 15522); $NiCl_2$ (Cat# 339350); $CuCl_2$ (Cat# 751944); $CaCl_2$ (Cat# C3306) were purchased from Sigma-Aldrich. All confocal microscopy images were acquired in µ-slide 18 well glass bottom chambers from ibidi (Cat# 81817), in some cases the chambers were coated with 0.1 mg/mL poly-*L*-lysine for 15 minutes to decrease the movement of the GUVs during imaging.

Supplementary Figures

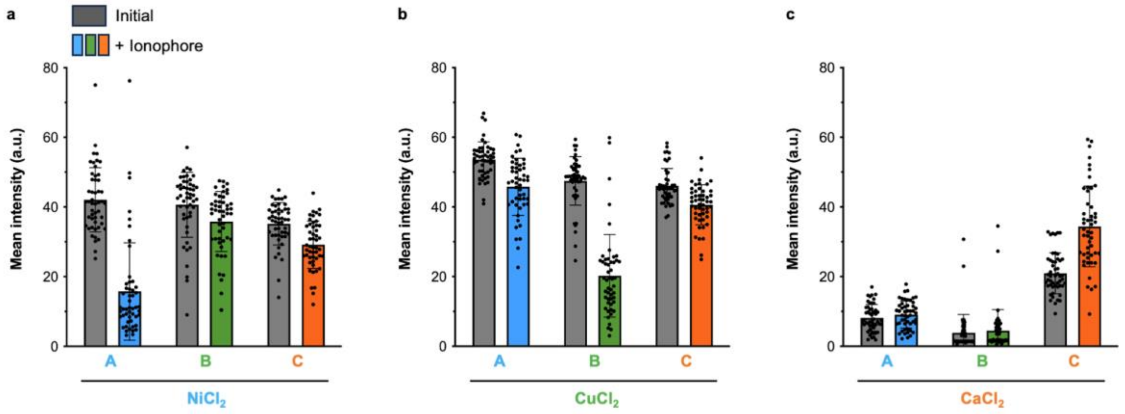

Figure S1. The mean Rhod2 fluorescence intensity inside GUVs in Fig. 1c-ii, which were used for the calculation of the transport efficiency in Fig. 1c-ii. Error bars represent the SD of the mean for. n_{GUV} = 50. **a**, Mean Rhod2 fluorescence intensity inside GUVs loaded with [Ca^{2+}-Rhod2] before and after addition of ionophore A, B or C in the presence of 1 µM external $NiCl_2$. **b**, Mean Rhod2 fluorescence intensity inside GUVs loaded with [Ca^{2+}-Rhod2] before and after addition of ionophore A, B or C in the presence of 1 µM external $CuCl_2$. **c**, Mean Rhod2 fluorescence intensity inside GUVs loaded with Rhod2 before and after addition of ionophore A, B or C in the presence of 100 µM external $CaCl_2$. Ionophore A, B and C were used at 20 µM, 5 µM and 1 µM, respectively.

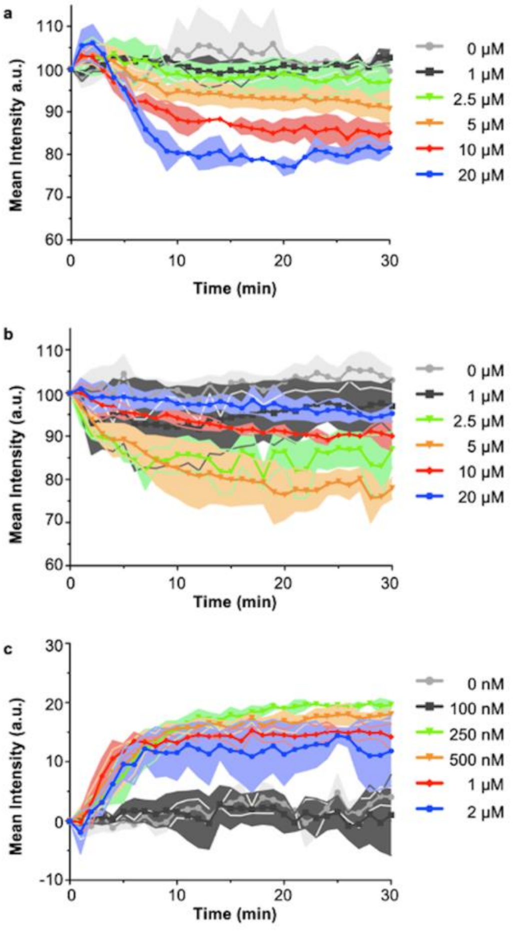

Figure S2. Timeline of metal transport into GUVs with varying ionophore concentration, measured by a plate reader. Mean fluorescence intensity of GUVs loaded with [Ca^{2+}-Rhod2] after addition of **a,** different concentrations of ionophore A in the presence of external $NiCl_2$; **b**, different concentrations of ionophore B in the presence of external $CuCl_2$. **c,** Mean fluorescence intensity of GUVs loaded with 100 μM $CaCl_2$ in the presence of external 2 μM Rhod2 after addition of different concentrations of ionophore C. Experiments were performed in triplicate and the error bars represent the SD of the mean intensities.

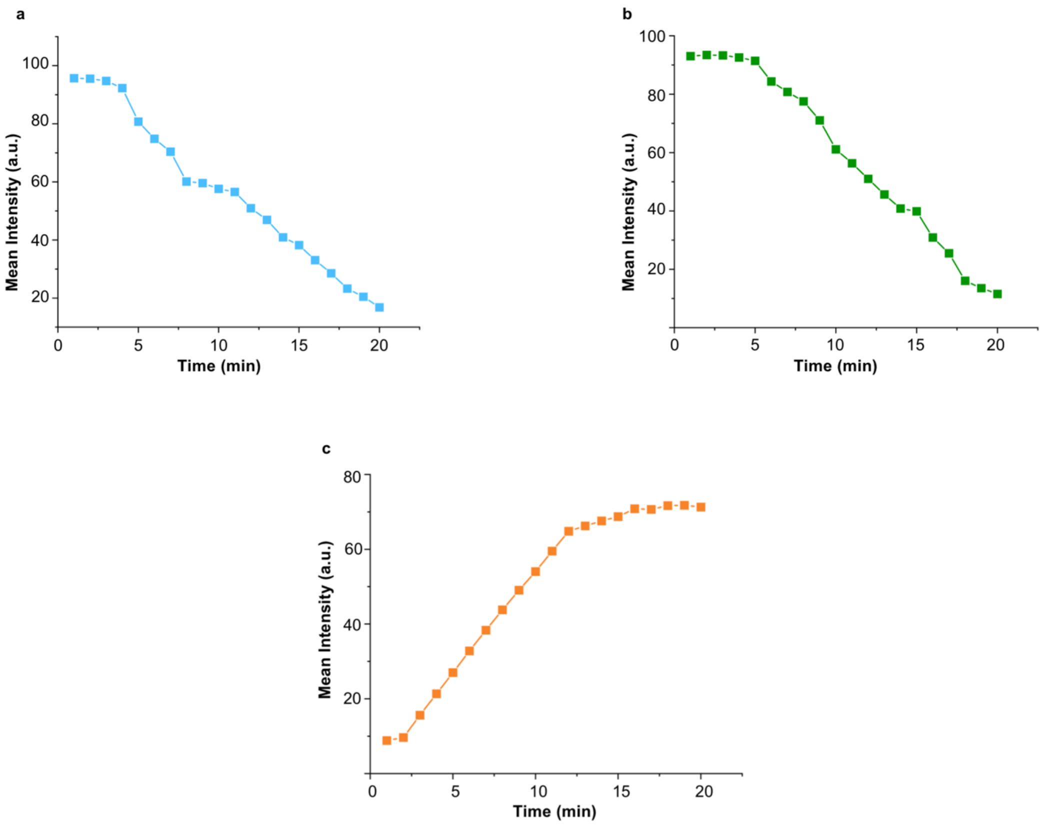

Figure S3. Timeline of metal transport into a single GUV tracked over time with different ionophores. Mean fluorescence intensity of a GUV loaded with [Ca^{2+}-Rhod2] after addition of **a,** ionophore A in the presence of external $NiCl_2$; or **b**, ionophore B in the presence of external $CuCl_2$. **c,** Mean fluorescence intensity of a single GUV tracked over time loaded with Rhod2 after addition of ionophore C in the presence of external $CaCl_2$.

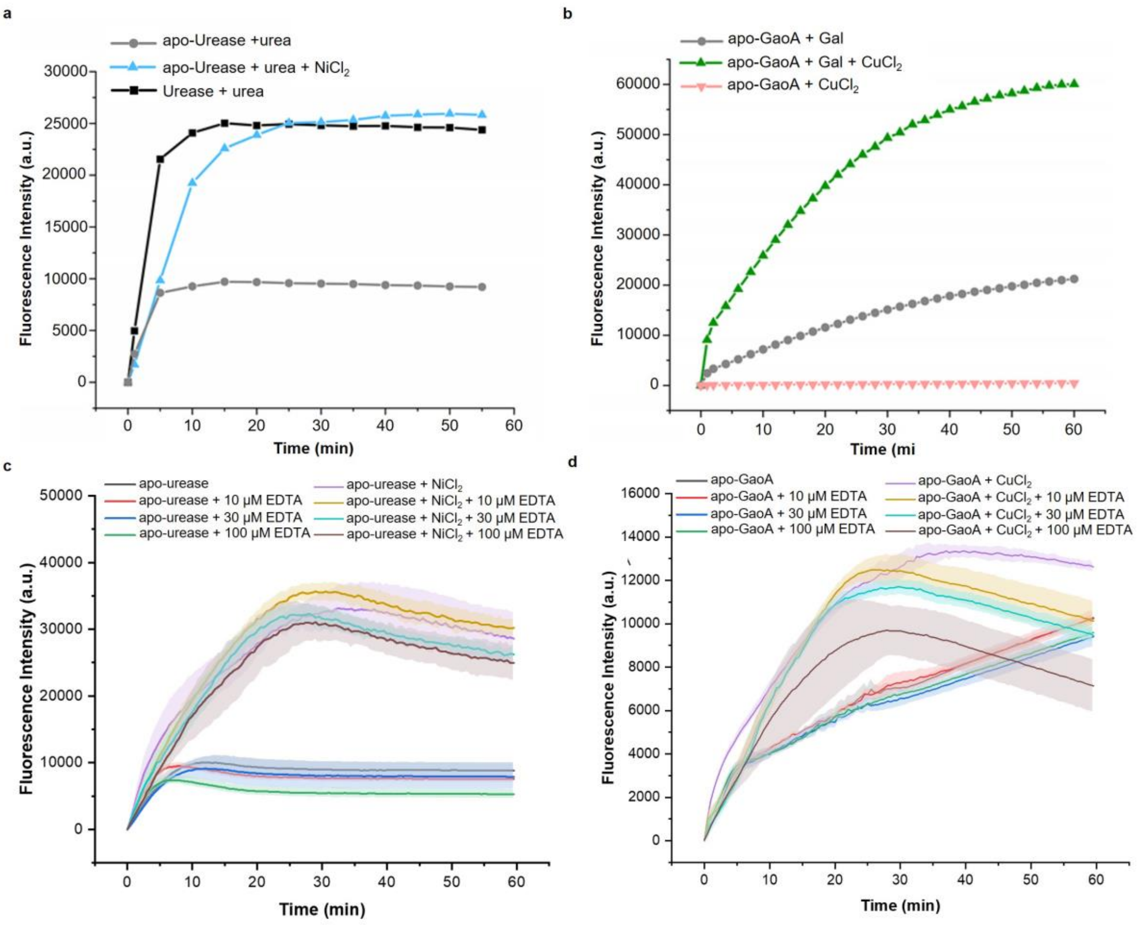

Figure S4. Timeline of apo-enzyme activation. a, Fluorescence of urease sensor in the presence of apo-urease in the presence and absence of $NiCl_2$ upon addition of urea (n = 1). **b**, Fluorescence of GaoA sensor in the presence of apo-GaoA in the presence and absence of $CuCl_2$ upon addition of galactose (n= 1). **c**, 1μM apo-urease in the presence of 10 mM urea activated with 1 μM $NiCl_2$ in the presence of varying concentrations of EDTA (n = 3, n is three parallel sample). The added EDTA has no effect on the urease activation. **d,** 150 nM apo-GaoA in the presence of 10 mM galactose activated with 1 μM $CuCl_2$ in the presence of varying concentrations of EDTA (n = 3, n is three parallel sample). 100 μM EDTA decreases the activation of GaoA. Error bars with fill area indicate the SD of the mean intensities.

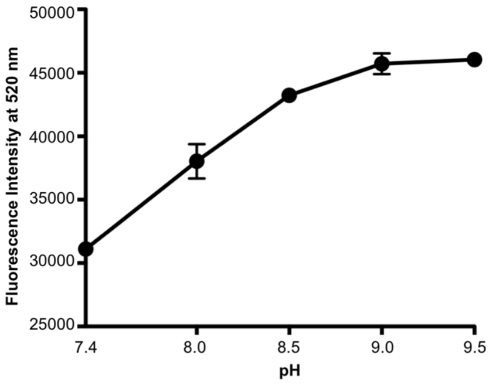

Figure S5. pH dependent fluorescence of the urease sensor as measured in solution. Urease sensor in Buffer A was divided into five aliquots, the pH was adjusted and the fluorescence intensity measured (λex = 488 nm; λem = 510-600 nm same as CLSM settings). The urease sensors changed its fluorescence up to pH 9. Experiments were performed in quadruplicates and the error bars represent the SD of the average intensities.

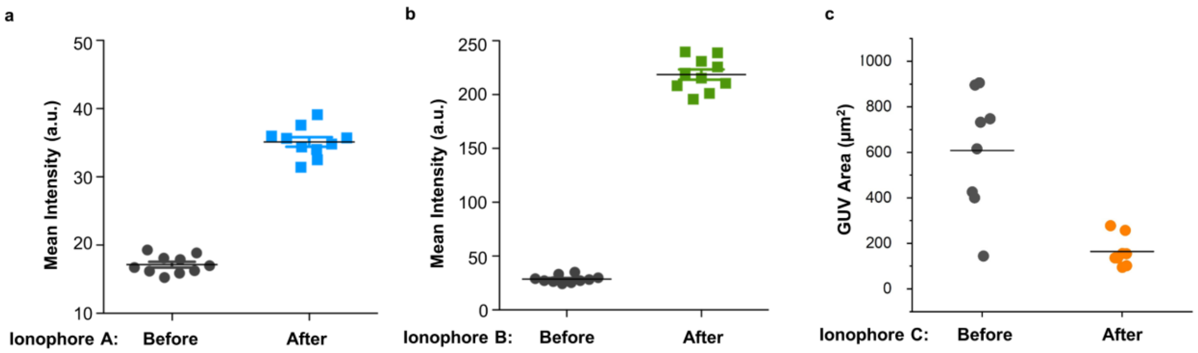

Figure S6. Comparison of the inactive and active states of each apo-enzyme. a, Mean fluorescence intensity of the urease sensor inside GUVs (n_{GUV} = 10) before and 20 min after addition of ionophore A. **b**, Mean fluorescence intensity of GaoA sensor inside GUVs (n_{GUV} = 10) before and 20 min after addition of ionophore B. **c**, Area of GUVs lysed before and after addition of ionophore C. (n_{GUV} = 8) Error bars represent the SD of the mean intensities.

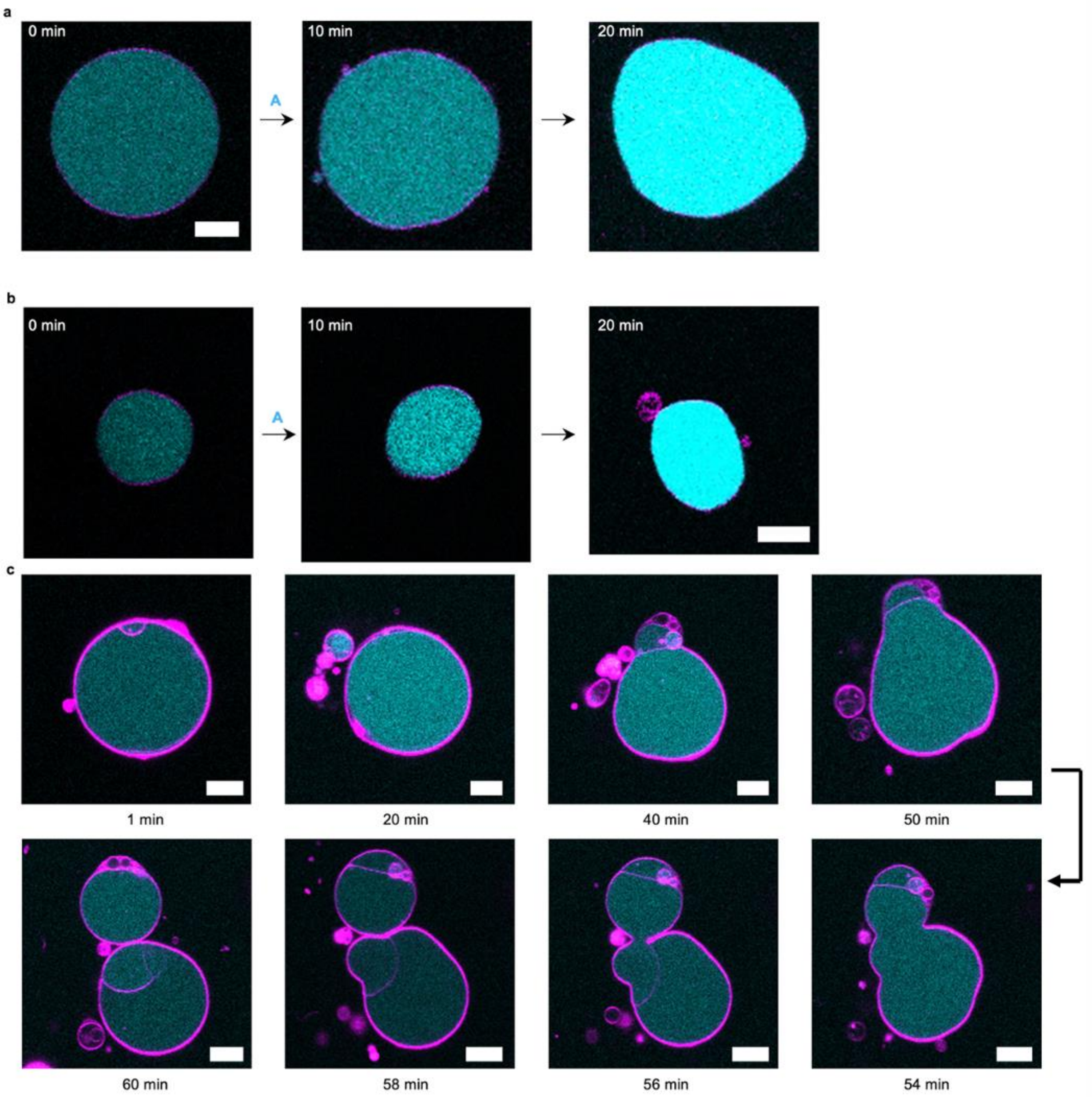

Figure S7. GUV deformation and interconnection as a result of elevated internal pH and osmotic pressure. Representative CLSM images of apo-urease loaded GUVs before and after addition of ionophore A in the presence of external $NiCl_2$: **a**, **b**, The intracellular activation of apo-urease resulted in the increase in intracellular pH, accompanied by the deformation of the GUV shape. Scale bar is 10 µm. **c**, In some GUVs, the intracellular activation of apo-urease also induced incomplete cell division (**Supplementary Movie 1**).

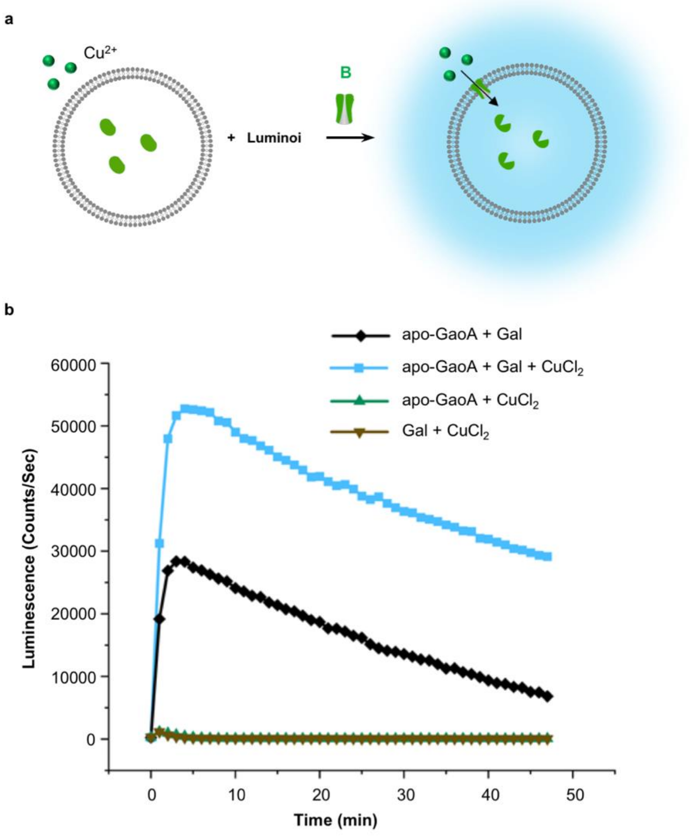

Figure S8. Activation of apo-GaoA inside GUVs with ionophore B shown with luminescence. **a**, Schematic representation of apo-GaoA and HRP loaded GUVs in the presence of luminol and external $CuCl_2$. The addition of ionophore B results in the generation of hydrogen peroxide, which reacts with luminol catalyzed by HRP and generates luminescence. **b**, Time-course of chemiluminescence intensities from the GUVs with apo-GaoA and galactose in the presence of external luminol, $CuCl_2$, and ionophore B. Luminescence intensity produced by apo-GaoA with galactose and $CuCl_2$ (blue line) is clearly higher than that produced by apo-GaoA with only galactose (black line), apo-GaoA with only $CuCl_2$ (green line), or no apo-GaoA with galactose and $CuCl_2$ (brown line). Measurements were started upon addition of luminol.

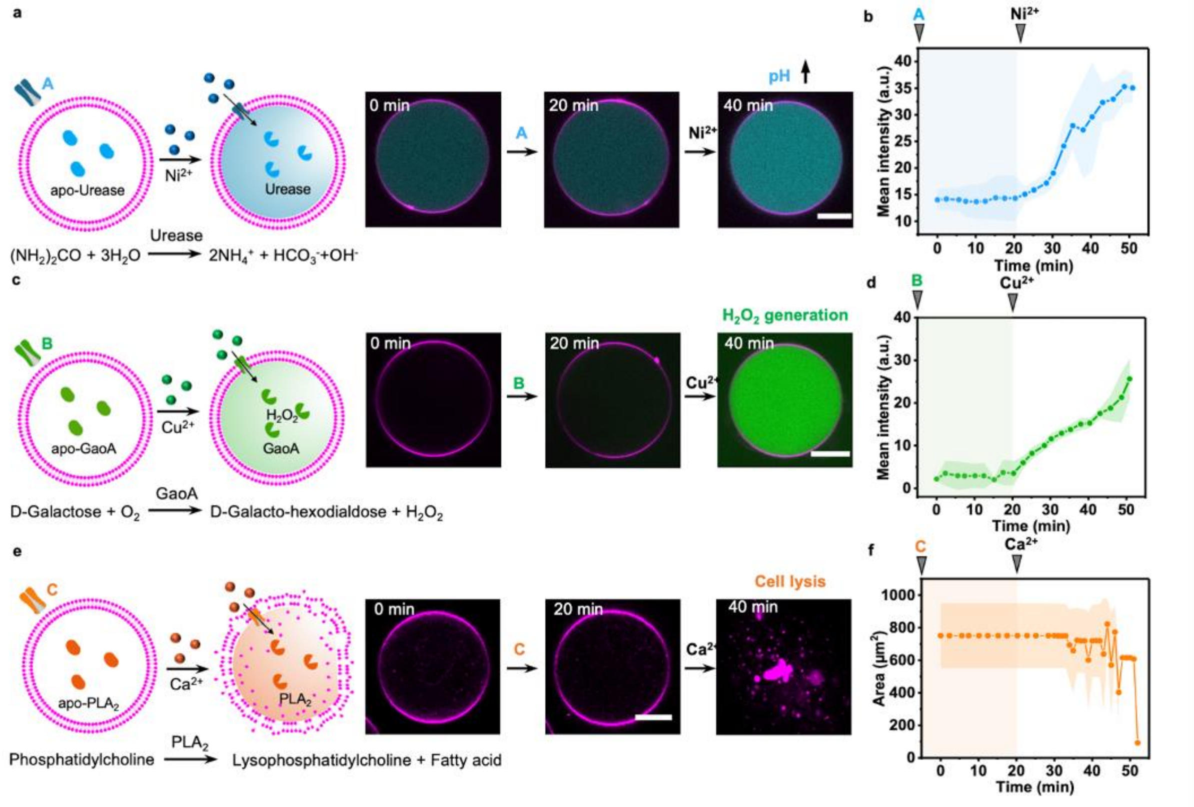

Figure S9. Adding the ionophore first and the metal ions seconds for activating apo-metalloenzymes in GUVs. a, Schematic and CLSM images of the same apo-urease loaded GUV (membrane shown in red) in the presence of ionophore A, increasing its intracellular pH (urease sensor shown in cyan) upon addition of Ni^{2+} due to Ni^{2+} transport into the GUV and activation of urease. b, Fluorescence increase inside GUVs with time in (a). n_{GUV} = 10. Error bars represent the SD. c, Schematic and CLSM images of the same apo-GaoA loaded GUV in the presence of ionophore B, producing H_2O_2 (GaoA sensor shown in green) upon addition of Cu^{2+} due to Cu^{2+} transport into the GUV and activation of GaoA. d, Fluorescence increase inside the GUV with time in (c). n_{GUV} = 10. Error bars represent the SD. e, Schematic and CLSM images of the same apo-PLA_2 loaded GUV, lysing upon addition of ionophore C due to Ca^{2+} transport into the GUV and activation of PLA_2. f, GUV area with time. n_{GUV} = 6. Error bars with fill area represent the SD of the average intensities. Scale bars are 10 μm.

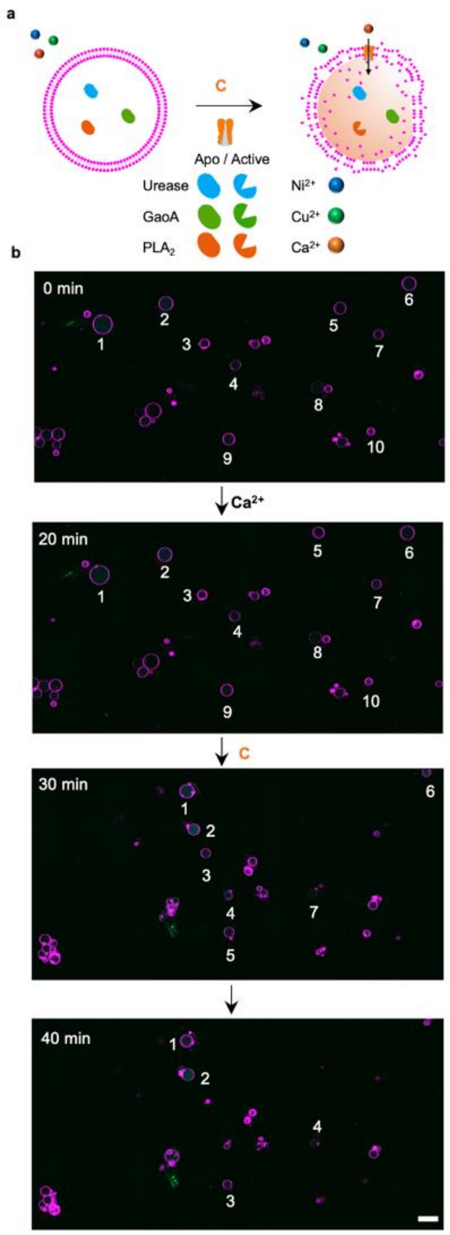

Figure S10. Response of urease and GaoA sensors upon addition of ionophore C. a, Schematic illustration of the GUVs loading all components. b, Representative merged CLSM images of GUVs loaded with all components, including three apo-metalloenzymes, substrates, urease (shown in cyan) and GaoA (shown in green) sensors in the presence of external $NiCl_2$, $CuCl_2$, $CaCl_2$. After the addition of Ca^{2+} and ionophore C after 20 min, the fluorescence of the urease and the GaoA sensors did not increase within 20 min. A decline in GUV numbers is observed concomitant with the activation of apo-PLA2 by ionophore C. Scale bars are 30 µm.

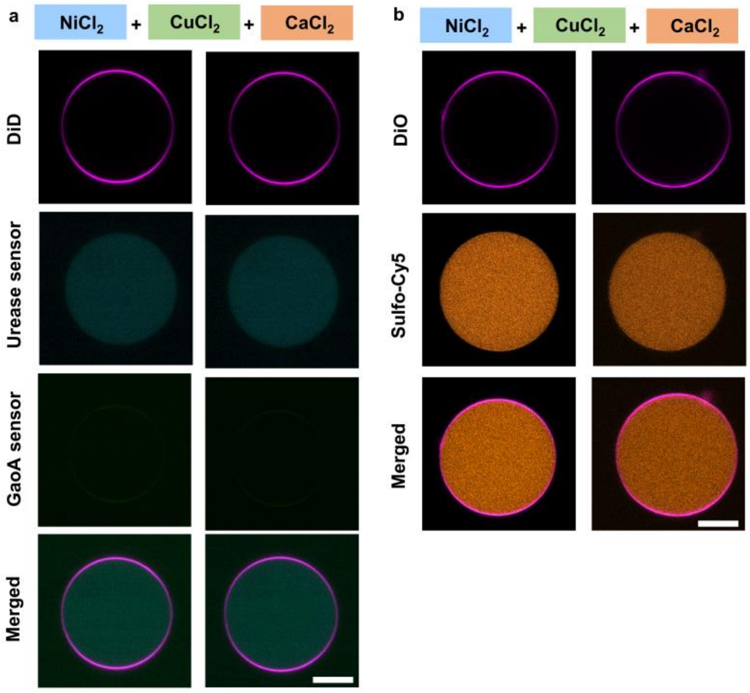

Figure S11. No activation of apo-enzymes in the presence of external metal ions. a, Representative CLSM images of GUVs loaded with three apo-metalloenzymes, substrates, urease (shown in cyan) and GaoA (shown in green) sensors in the presence of external all three metal ions. The fluorescence of neither the urease nor the GaoA sensor increase after 60 min incubation. **b,** Representative CLSM images of GUVs loaded with three apo-metalloenzymes, substrates and sulfo-Cy5 (shown in orange) in the presence of external all three metal ions. The GUVs remain stable and don't leak after 60 min. GUV membrane dye shown in red. Scale bars are 10 µm.

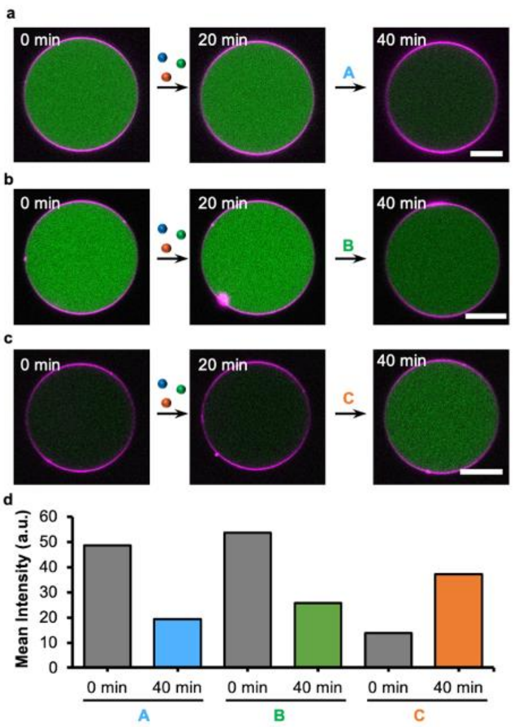

Figure S12. Ionophore activity in the presence of all three metal ions. Representative CLSM images of GUVs loaded with [Ca^{2+}-Rhod2] (in a and b) or Rhod2 (in c) before and after addition of **a.** ionophore A, **b,** ionophore B, or **c,** ionophore C in the presence of all three metal ions. **d,** Quantification of Rhod2 fluorescence intensity in individual GUVs shown in panels a, b, and c at 0 and 40 minutes. Scale bars are 10 µm. The presence of the other metal ions does not disturb ion transport by the cognate ionophore.

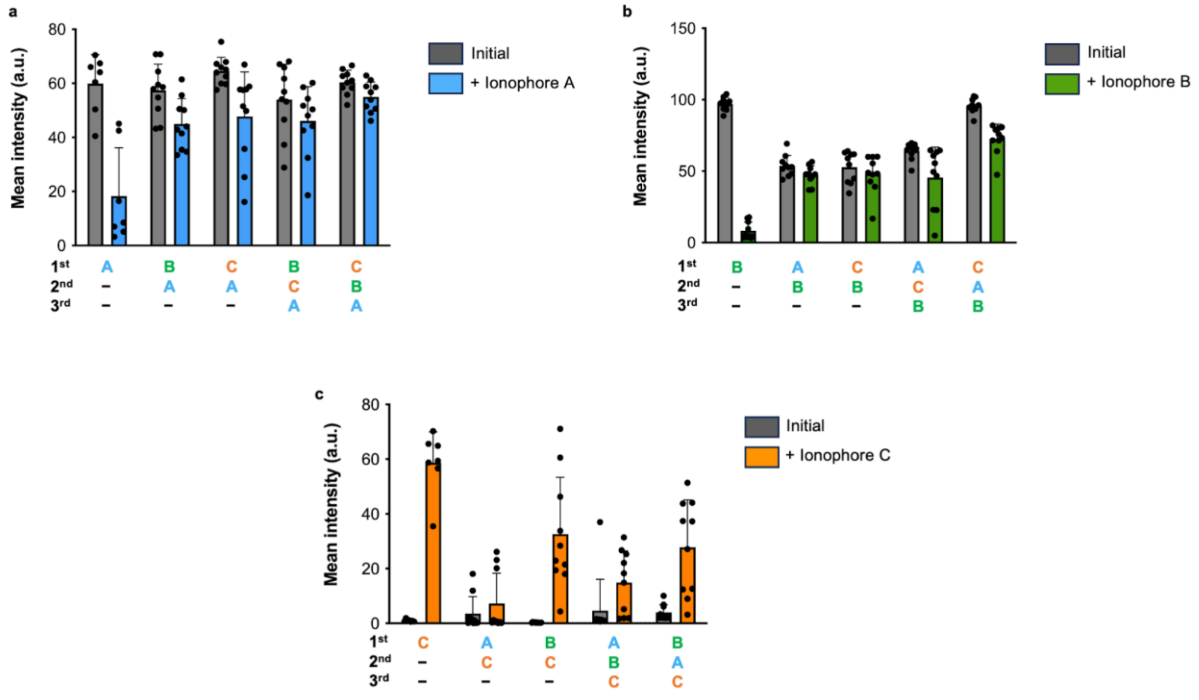

Figure S13. Bar graphs show the negative influence of prior ionophores on transport with subsequent ionophores. The mean Rhod2 fluorescence intensity inside GUVs in Fig. 4a, c, e, which were used for the calculation of the transport efficiency in Fig. 4b, d, f. **a**, Mean Rhod2 fluorescence intensity inside GUVs loaded with [Ca^{2+}-Rhod2] before and after addition of multiple ionophores (20 µM A, 5 µM B and 1 µM C) with 20 min intervals in the presence of external $NiCl_2$. **b**, Mean Rhod2 fluorescence intensity inside GUVs loaded with [Ca^{2+}-Rhod2] before and after addition of multiple ionophores (20 µM A, 5 µM B and 1 µM C) with 20 min intervals in the presence of external $CuCl_2$. **c**, Mean Rhod2 fluorescence intensity inside GUVs loaded with Rhod2 before and after addition of multiple ionophores (20 µM A, 5 µM B and 1 µM C) with 20 min intervals in the presence of external $CaCl_2$.. Error bars represent the SD of the average intensities for n_{GUV} = 10.

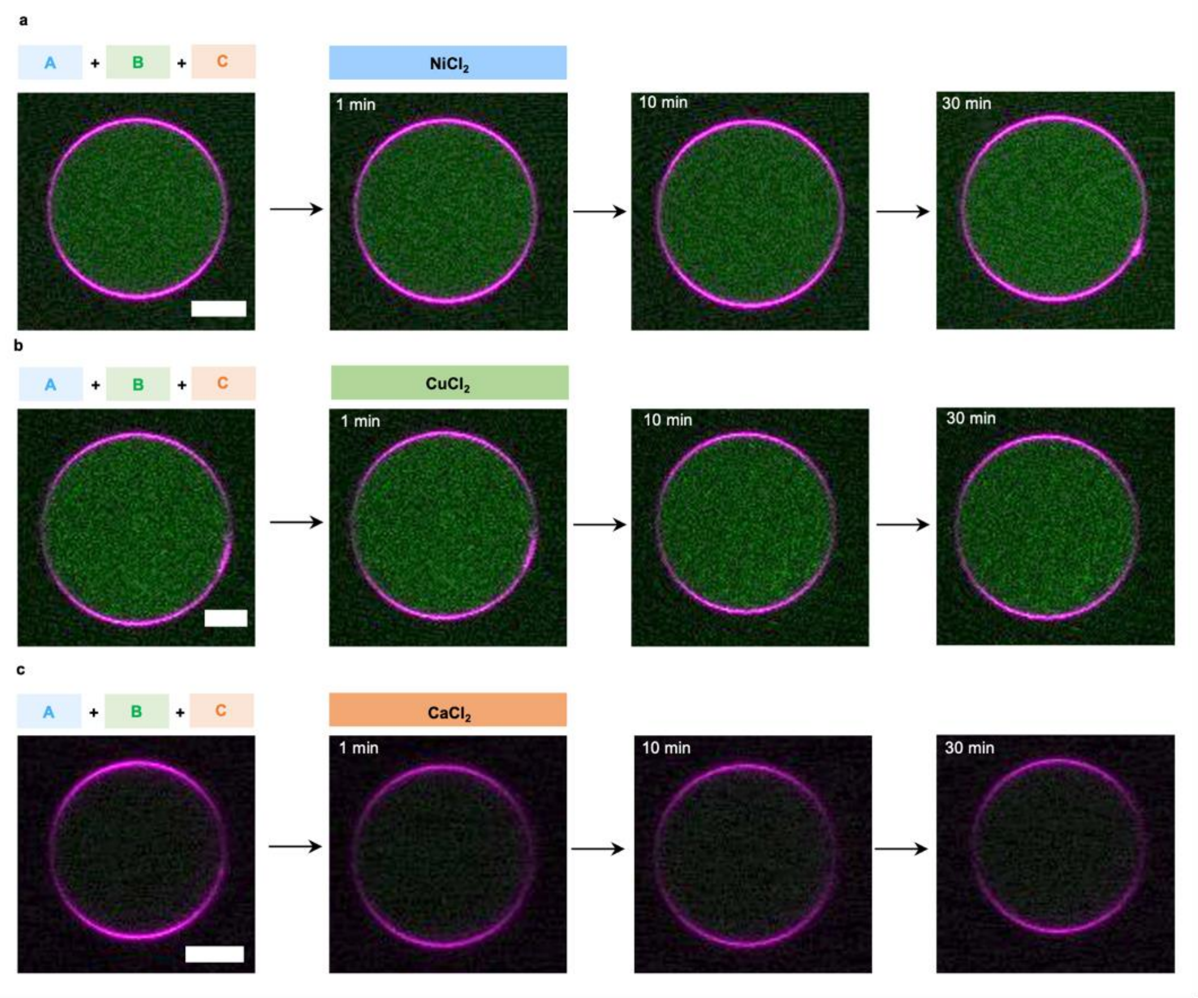

Figure S14. Adding all three ionophores at once induced no change of Rhod2 fluorescence in the presence of external metal ions. Representative CLSM images of [Ca^{2+}-Rhod2] loaded GUVs before and after addition all three ionophores in the presence of **a,** Ni^{2+}, or **b,** Cu^{2+} at different time points. **c**, Representative CLSM images of Rhod2 loaded GUV before and after addition all three ionophores in the presence of Ca^{2+}. Scale bars are 10µm.

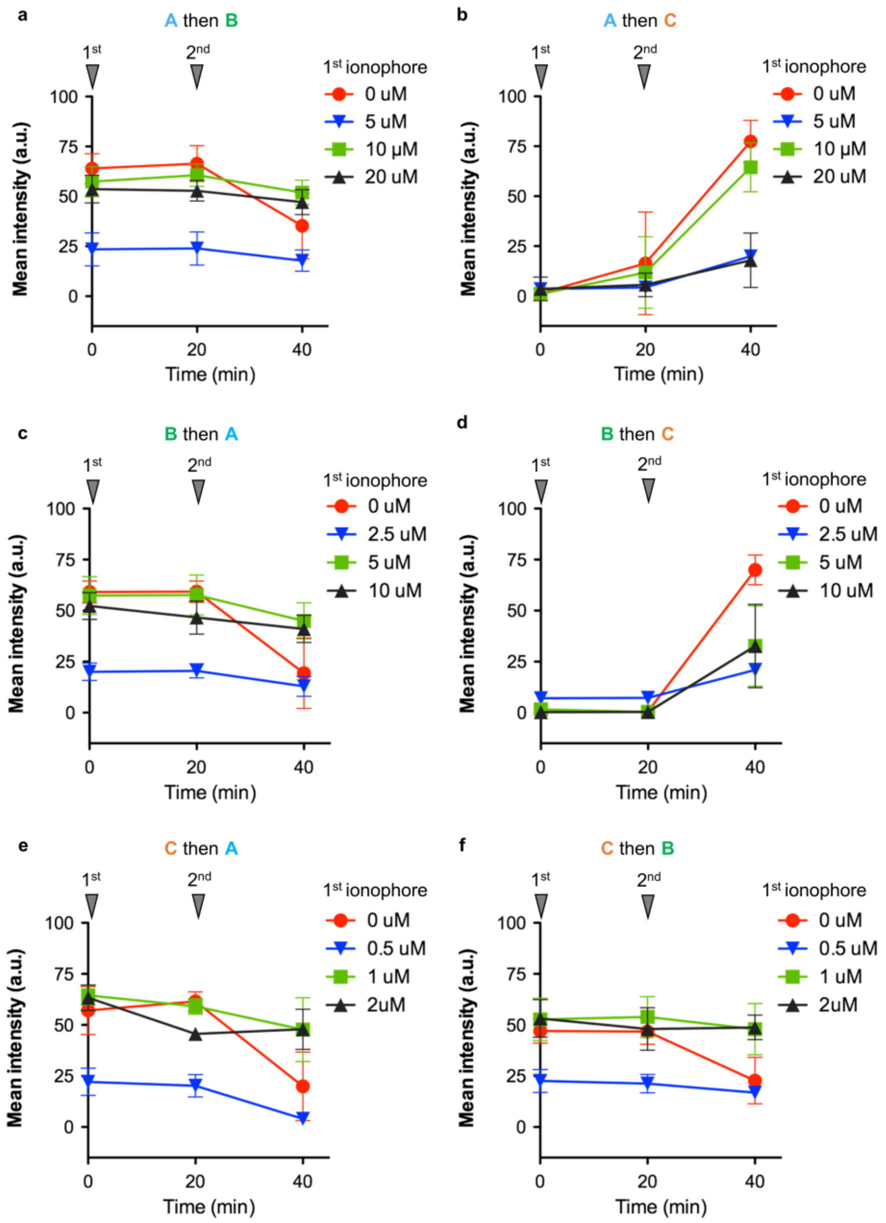

Figure S15. Prior ionophore concentration affects its negative impact on subsequent ionophore transport. Mean [Ca^{2+}-Rhod2] fluorescence intensity inside GUVs in CLSM images, which were used to calculate transport efficiency in Fig. 4g. GUVs were first incubated with different concentration of ionophore A and 20 min later with **a,** ionophore B in the presence of external $CuCl_2$ or **b,** ionophore C in the presence of external $CaCl_2$. GUVs were first incubated with different concentration of ionophore B and 20 min later with **c,** ionophore A in the presence of external $NiCl_2$ or **d,** ionophore C in the presence of external $CaCl_2$. GUVs were first incubated with different concentration of ionophore C and 20 min later with **e,** ionophore A in the presence of external $NiCl_2$ or **f,** ionophore B in the presence of external $CuCl_2$. GUVs in a, c, e and f were loaded with [Ca^{2+}-Rhod2] and in b and d with Rhod2. n_{GUV} = 10 for each data point. Error bars represent the SD.

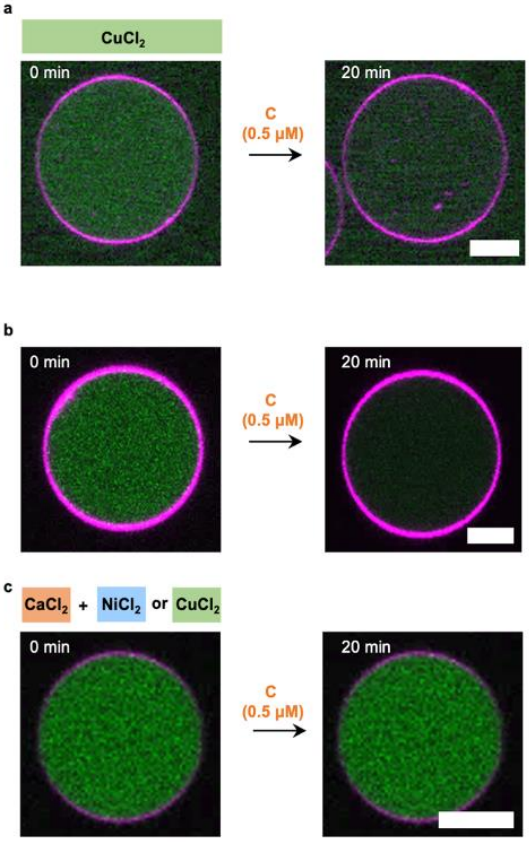

Figure S16. The diffusion out of GUVs depends on concentration gradient between the inside and the outside. Representative CLSM images of GUV loaded with [Ca^{2+}-Rhod2] before and after addition of 0.5 µM ionophore C **a,** in the presence of external $CuCl_2$; **b,** in the absence of external metal ion; **c,** in the presence of external $CaCl_2$ and $NiCl_2$ or $CuCl_2$. In (a) and (b) Ca^{2+} ions diffuse out of the GUV, resulting in a lower internal fluorescence. In (c) the internal and external Ca^{2+} concentrations were equal resulting in no change of [Ca^{2+}-Rhod2] fluorescence inside the GUV. Scale bars are 10 µm.

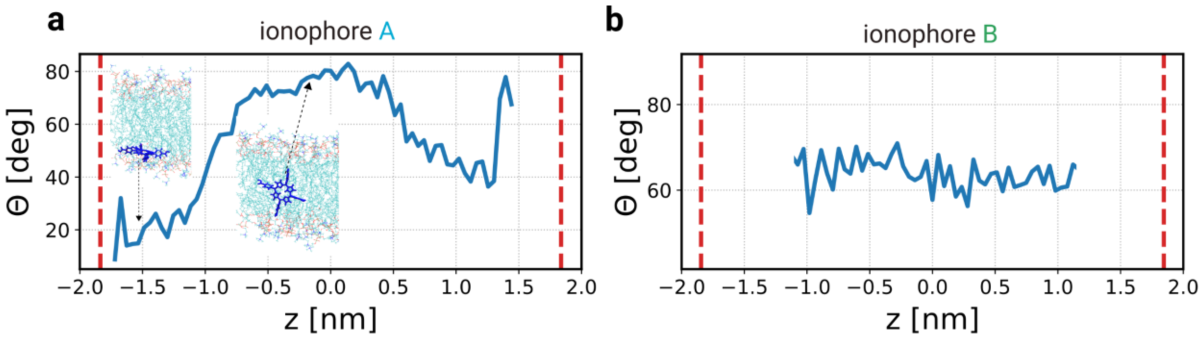

Figure S17. Orientation of the ionophores inside the membrane. a, The angle between the vector perpendicular to the ring part of ionophore A and **b**, ionophore B with the membrane normal (z-axis) as a function of the position of the ionophore inside the membrane. The average z-positions of the COM of lipids head group in the two leaflets are represented by red dashed lines.

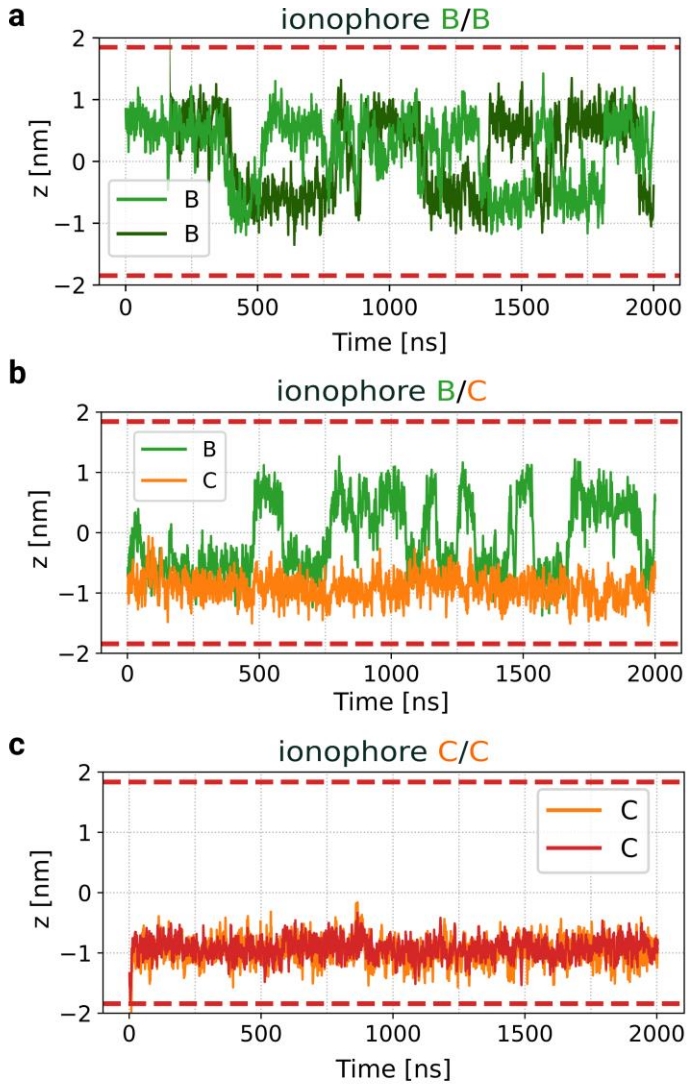

Figure S18 Two ionophores in the membrane. a, The time-dependent positions of the z-coordinates of two ionophores B, defined along the membrane normal of the COM. **b**, Similar to a) but with $N_B = N_C = 1$. **c**, Similar to a) but with $N_C = 2$. The position of the center of the membrane is at z = 0 and the approximate average positions of the lipids head group in the two leaflets are represented by red dashed lines.

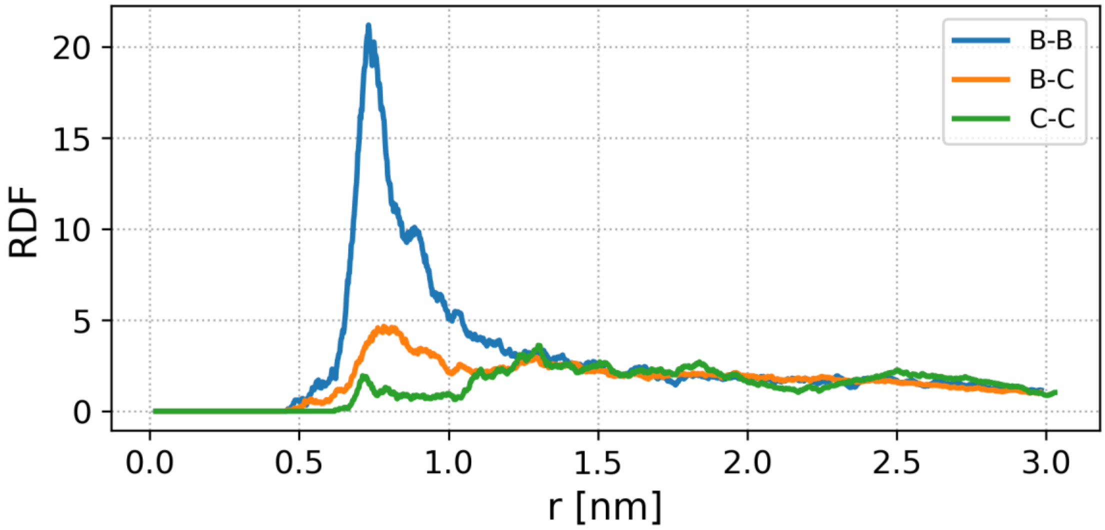

Figure S19 Radial distribution functions. The RDFs for the systems with NB=2, NB= NC=1, NC =2, respectively. It complements the RDFs shown in Fig.5h.

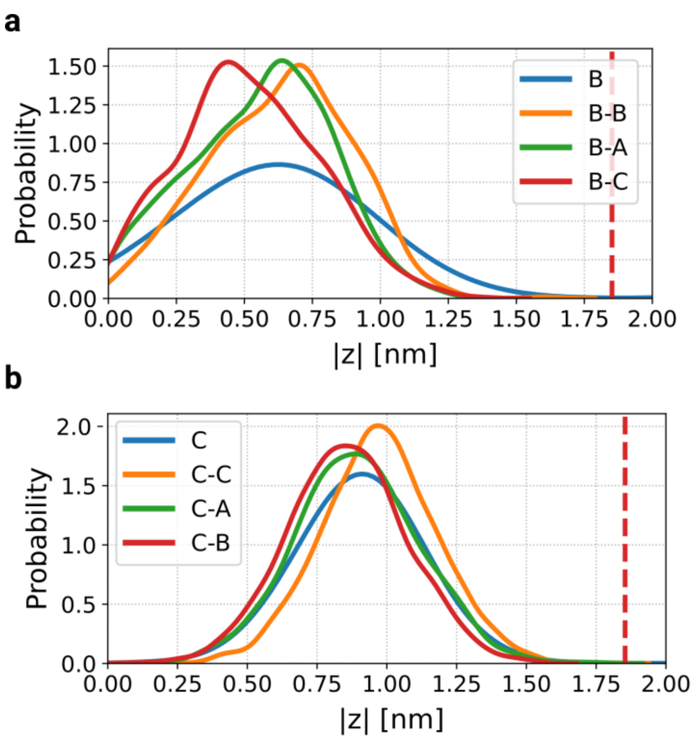

Figure S20 Height distribution of ionophores. a, The probability of finding ionophore B at a specific z value in the intensity-sensitive representation, see methods. For the case of two ionophores only those snapshots contribute where both ionophores are in the same leaflet. **b,** Similar to a) for the C ionophore.

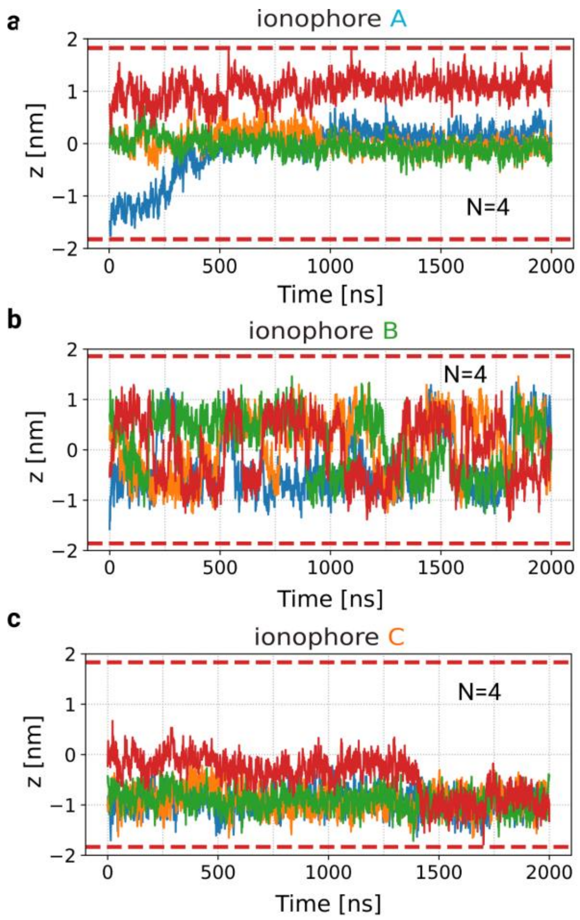

Figure S21 Many ionophores in the membrane. The time-dependent positions of the z-coordinates of four ionophores: **a,** N_A=4, **b,** N_B=4, **c,** N_C=4.

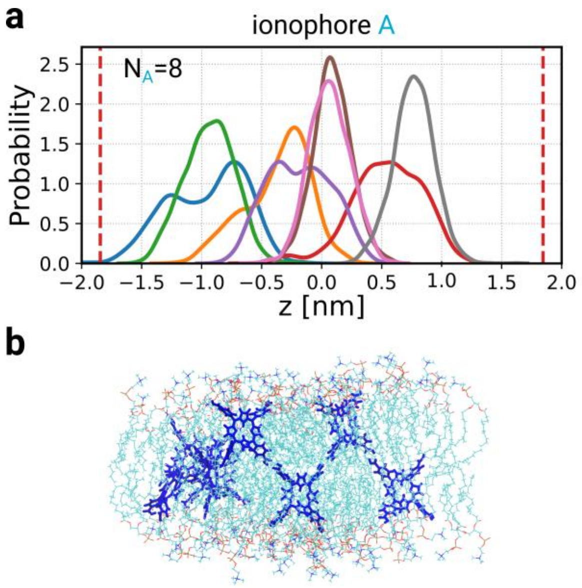

Figure S22 Distribution of individual ionophores in the membrane. a, The time-dependent positions of the z-coordinates in a system with N_A=8 of all eight ionophores, each ionophore represented as a different colored line. The ionophores either stay in the center of the membrane or in a single leaflet during the whole simulation time. **b,** A snapshot of that system, showing the clustering behavior as well as the specific interaction motif of ionophores in different leaflets.

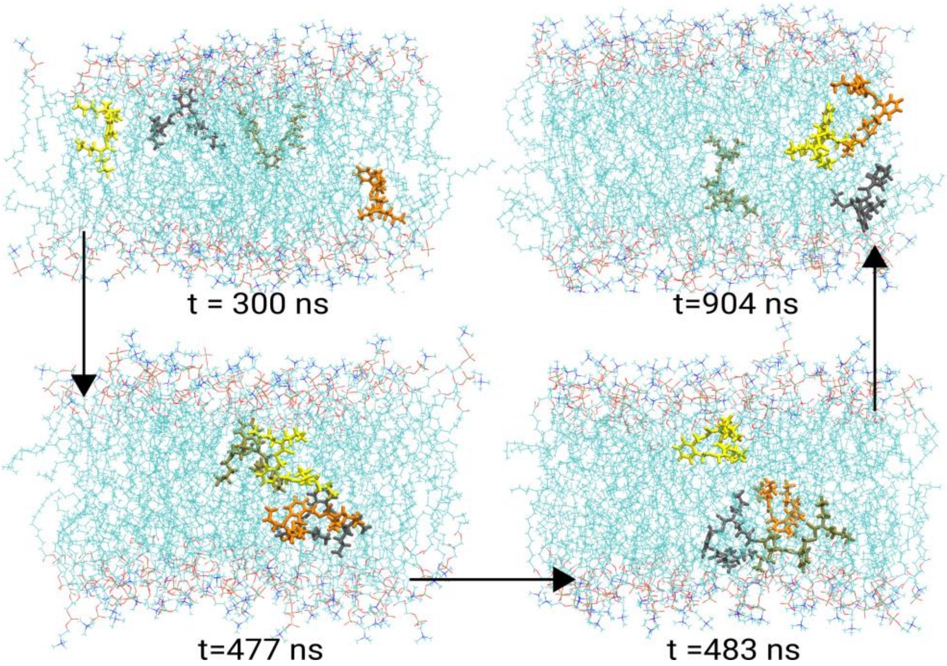

Figure S23. Snapshots of the ionophores B in N_B=4. The snapshots of ionophore B shows different configurations and interactions between them and building of transient clusters.

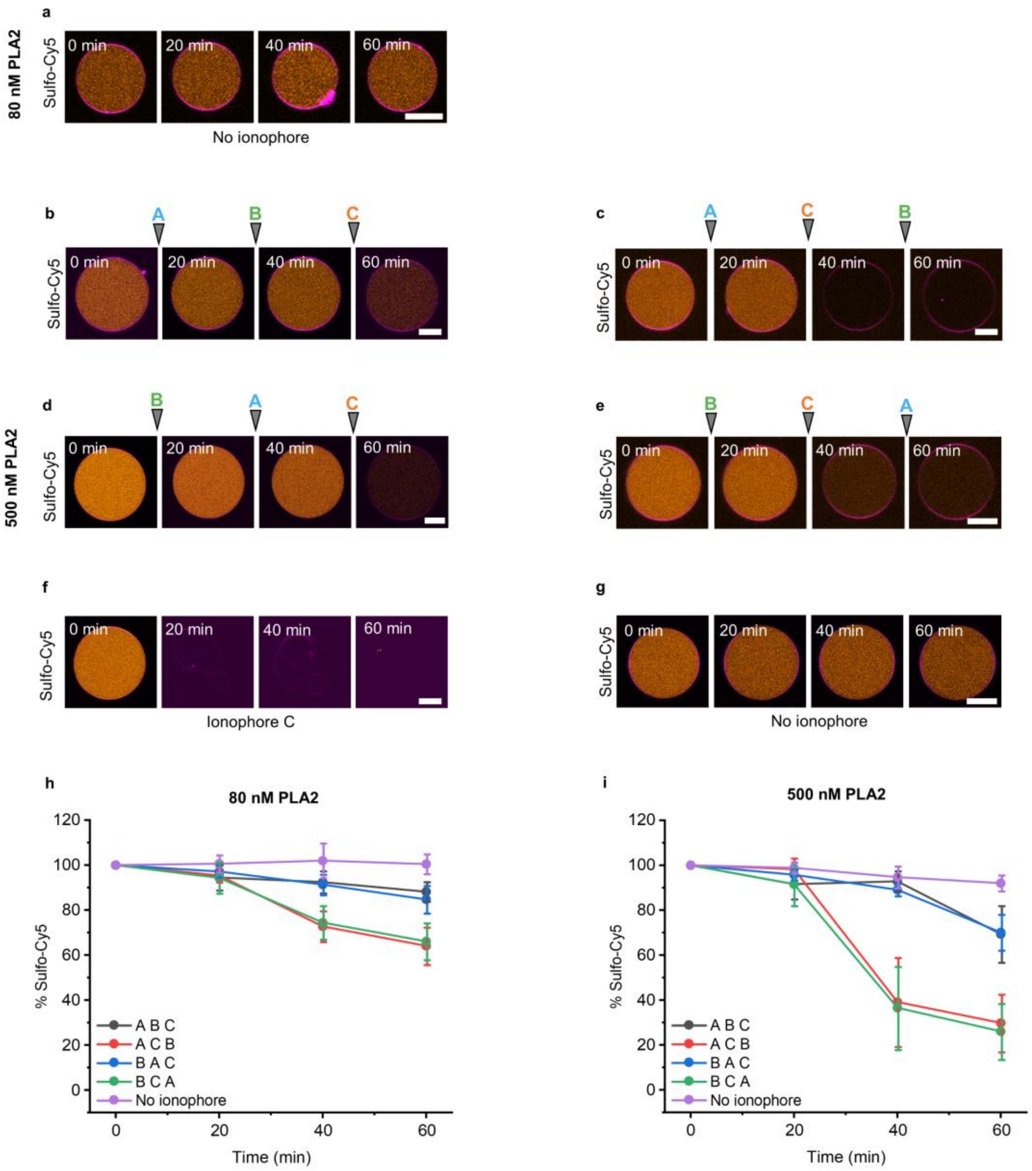

Figure S24. Permeability of GUVs after sequential addition of ionophores due to a partial activation of apo-PLA$_2$ with ionophore C transported Ca^{2+}. a, Representative CLSM images of GUVs loaded with sulfo-Cy5 and three apo-metalloenzymes (80 nM apo-PLA$_2$) in the presence of all three metal ions and the absence of ionophore C. Scale bars are 10 µm. **b-g,** Representative CLSM images of GUVs loaded with sulfo-Cy5 and three apo-metalloenzymes (500 nM apo-PLA$_2$) in the presence of all three metal ions and different ionophores added with 20 min intervals. Scale bars are 10 µm. Mean fluorescence intensity of the sulfo-Cy5 inside the GUVs (n_{GUV} = 10) after the sequential addition of multiple ionophores shown in **h,** (a and Fig. 6) with 80 nM PLA$_2$ and **i,** (b-g) with 500 nM PLA$_2$. Error bars represent the SD.

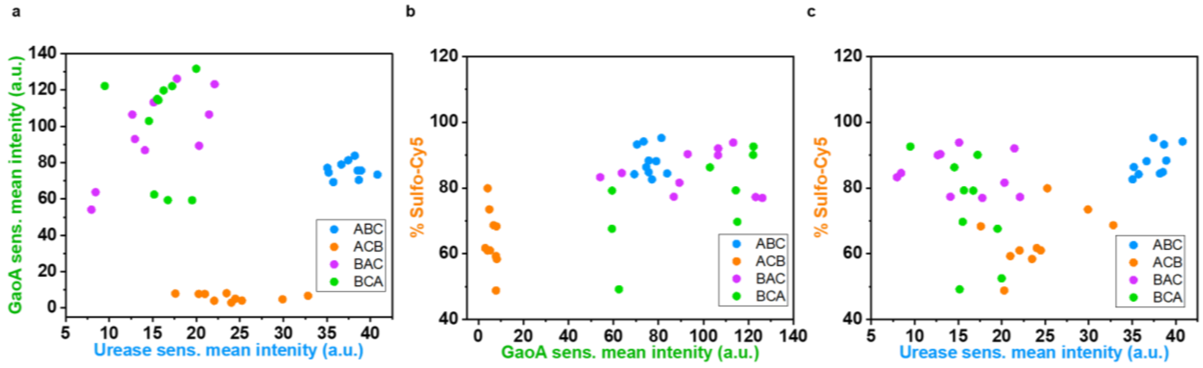

Figure S25 Two-D plots showing final states in each cell fate. a, The final mean intensity of **a,** urease and GaoA sensor, **b,** urease sensor and sulfo-Cy5 and **c,** GaoA sensor and sulfo-Cy5 after sequential addition of the three ionophores. Each GUV (n_{GUV}= 10 in each condition) is shown as a single dot.

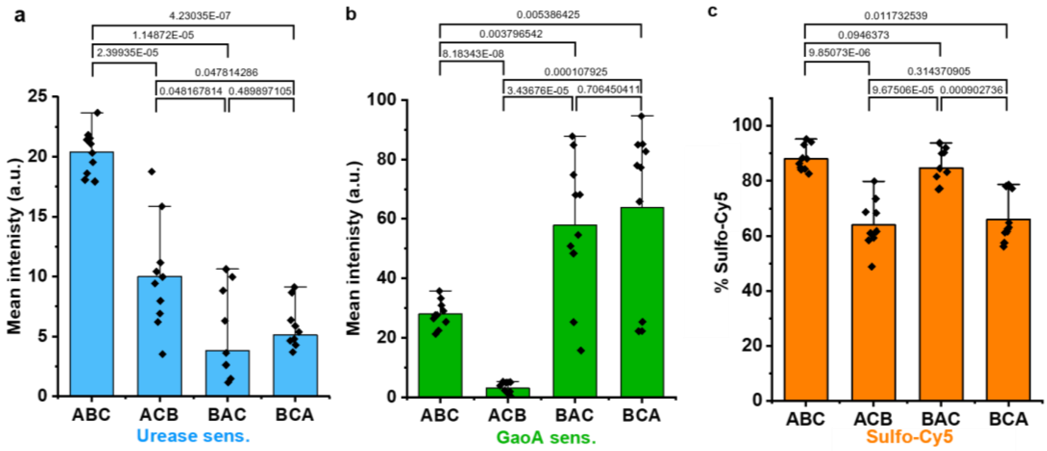

Figure S26 Bar graphs for the final states after the sequential addition of three ionophores. The final mean intensity of **a,** urease sensor, **b,** GaoA sensor, and **c,** % sulfo-Cy5 after sequential addition of the three ionophores. Error bars represent SD of the average intensities for n_{GUV}=10.

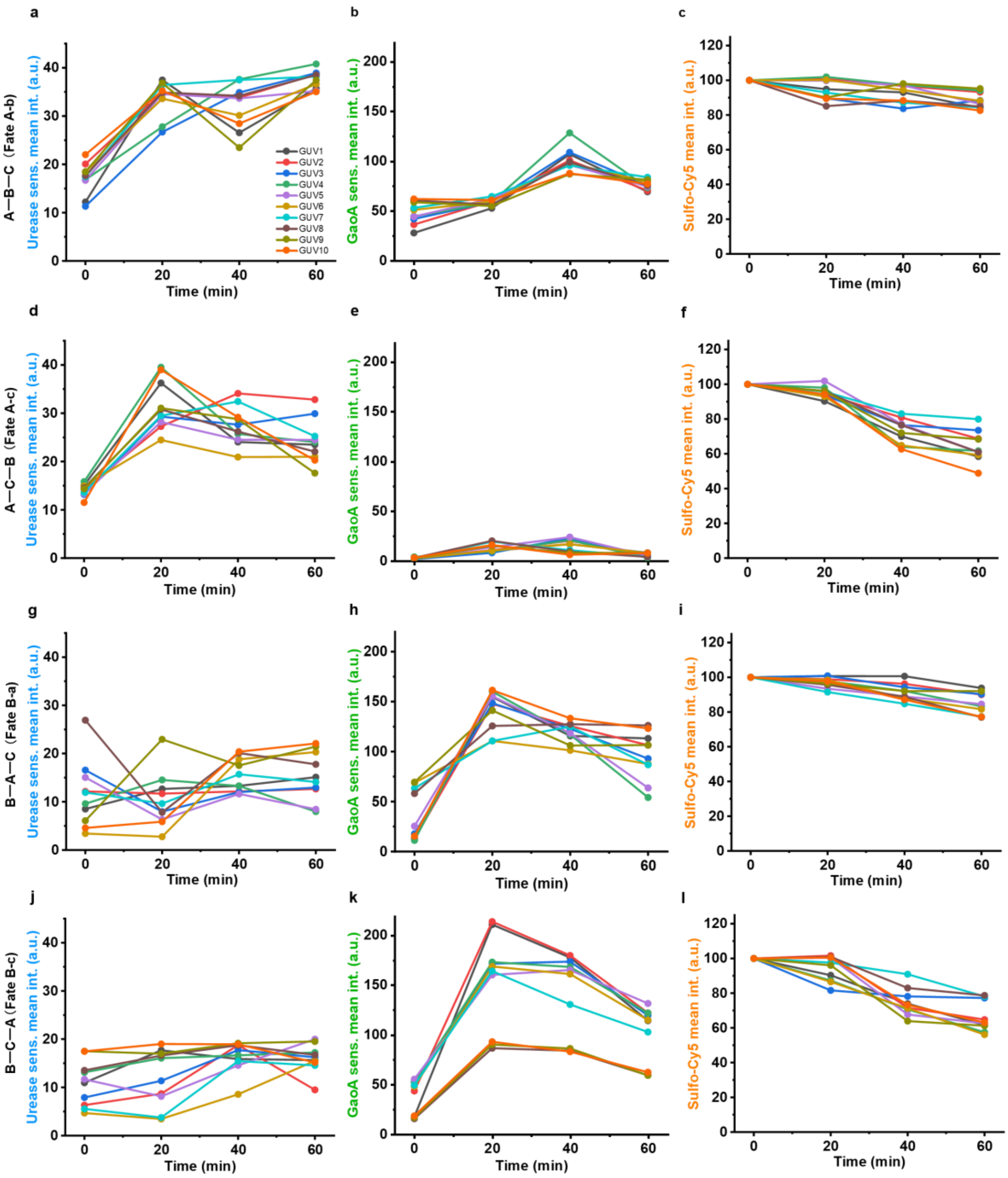

Figure S27 Timelines for individual GUVs. a, d, g, j urease sensor, **b, e, h, k** GaoA sensor, and **c, f, i, l** % sulfo-Cy5 after sequential addition of the three ionophores indicated on the left side.

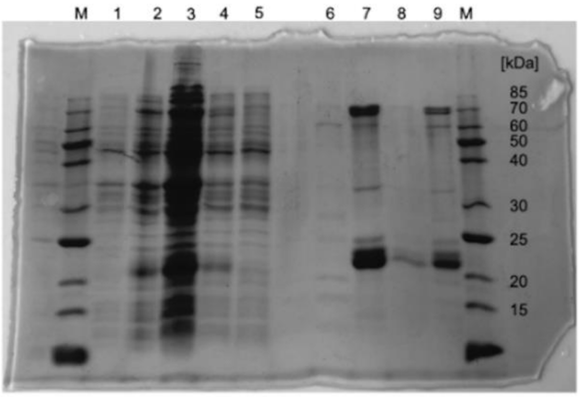

Figure S28 12% SDS-PAGE gel performed to analyze the fractions in purifying apo-GaoA. Cells were lysed and pelleted fractions were analyzed by SDS-PAGE and Coomassie blue staining. M, Marker, Lane 1, uninduced; Lane 2, induced; Lane 3, Pelleted; Lane 4, Lysate; Lane 5, Flow through; Lane 6, wash; Lane 7-8, Elutions; Lane 9, Concentrated GaoA solution.
